# Supplementary figures and images for: A critical role for heme synthesis and succinate in the regulation of pluripotent states transitions
Source: eLife. 2023 Jul 10;12:e78546. doi: 10.7554/eLife.78546 (PMC10425175; doi:10.7554/eLife.78546)

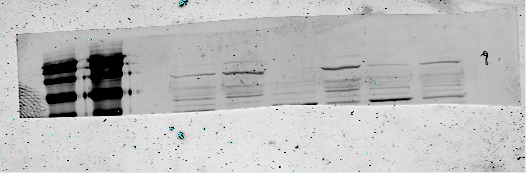

Supplement: Figure 1—source data 1. [file elife-78546-fig1-data1.zip › Figure 1 - Source files/Fig 1 source - DNMT3A.jpg]

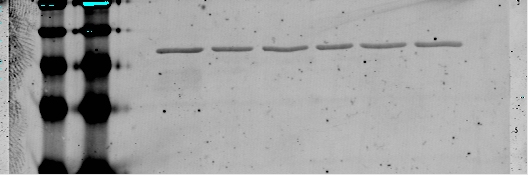

Supplement: Figure 1—source data 1. [file elife-78546-fig1-data1.zip › Figure 1 - Source files/Fig 1 source - GAPDH 2.jpg]

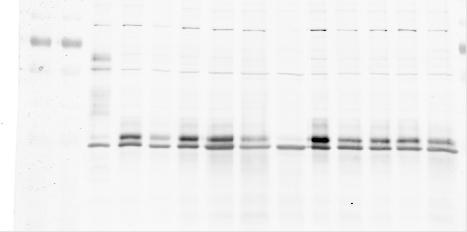

Supplement: Figure 1—source data 1. [file elife-78546-fig1-data1.zip › Figure 1 - Source files/Fig 1 source - OTX2.jpg]

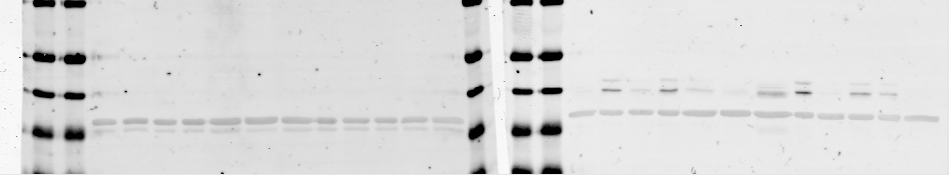

Supplement: Figure 1—source data 1. [file elife-78546-fig1-data1.zip › Figure 1 - Source files/Fig 1 source GAPDH.jpg]

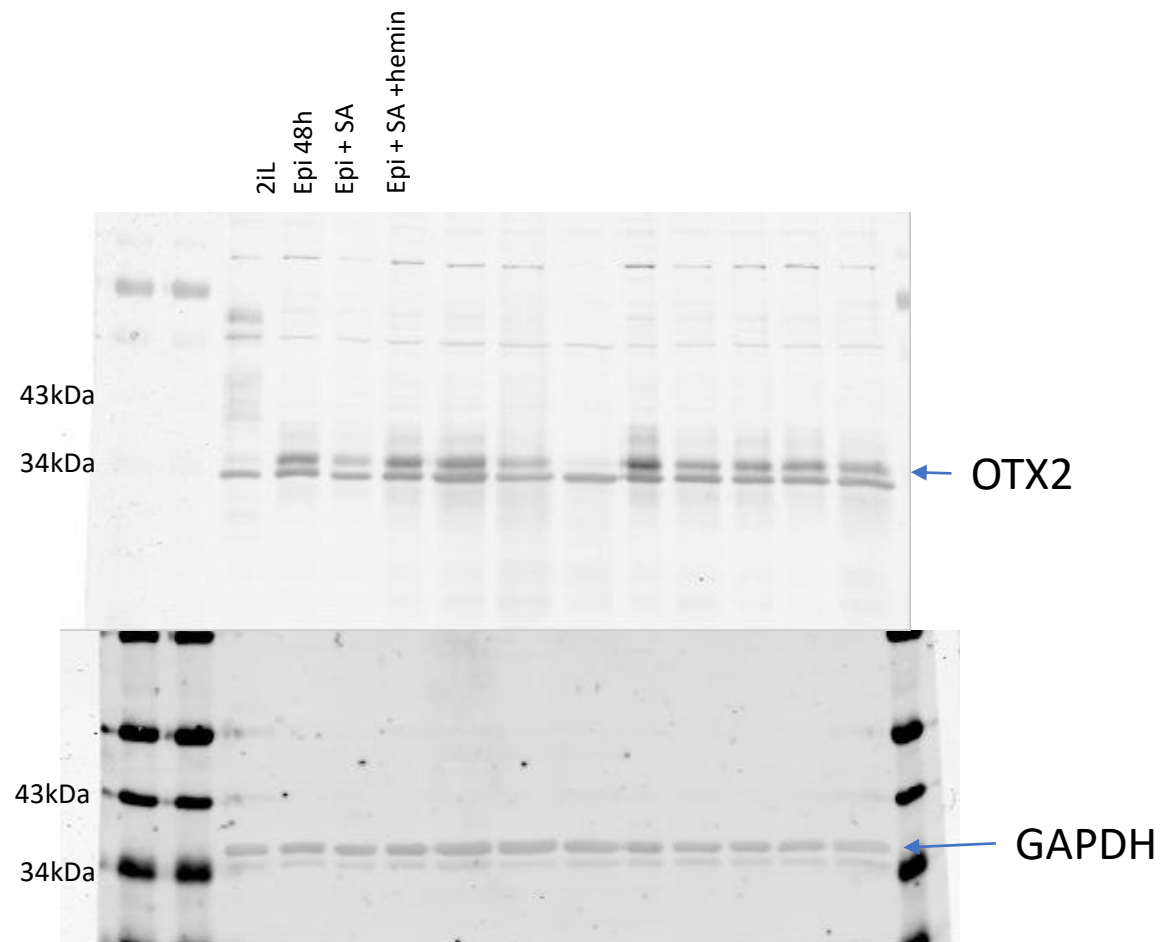

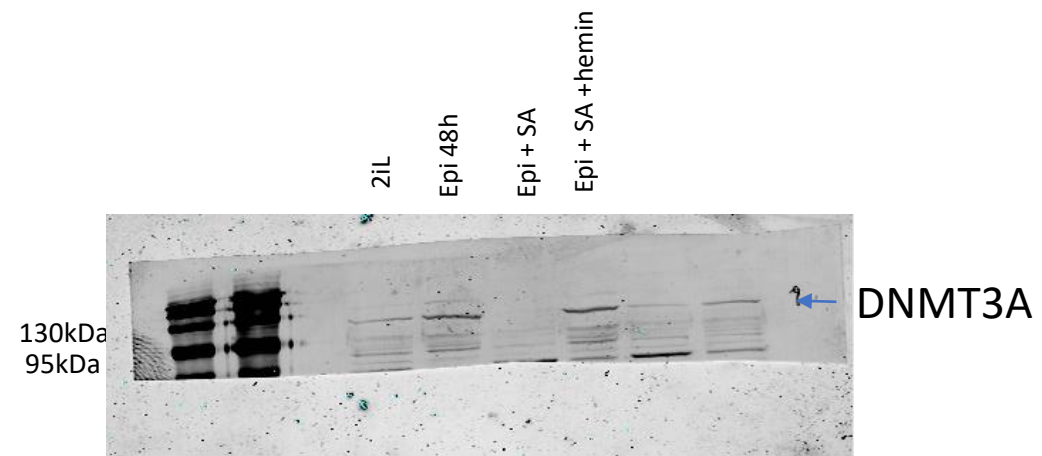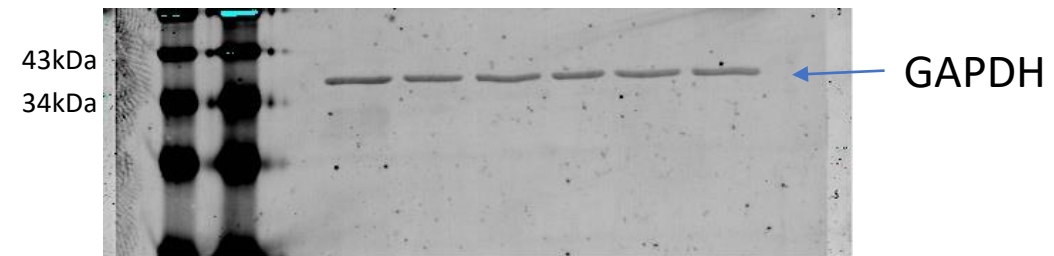

Supplement: Figure 1—source data 1. [file elife-78546-fig1-data1.zip › Figure 1 - Source files/figure 1 - source files - Annotated.pdf]

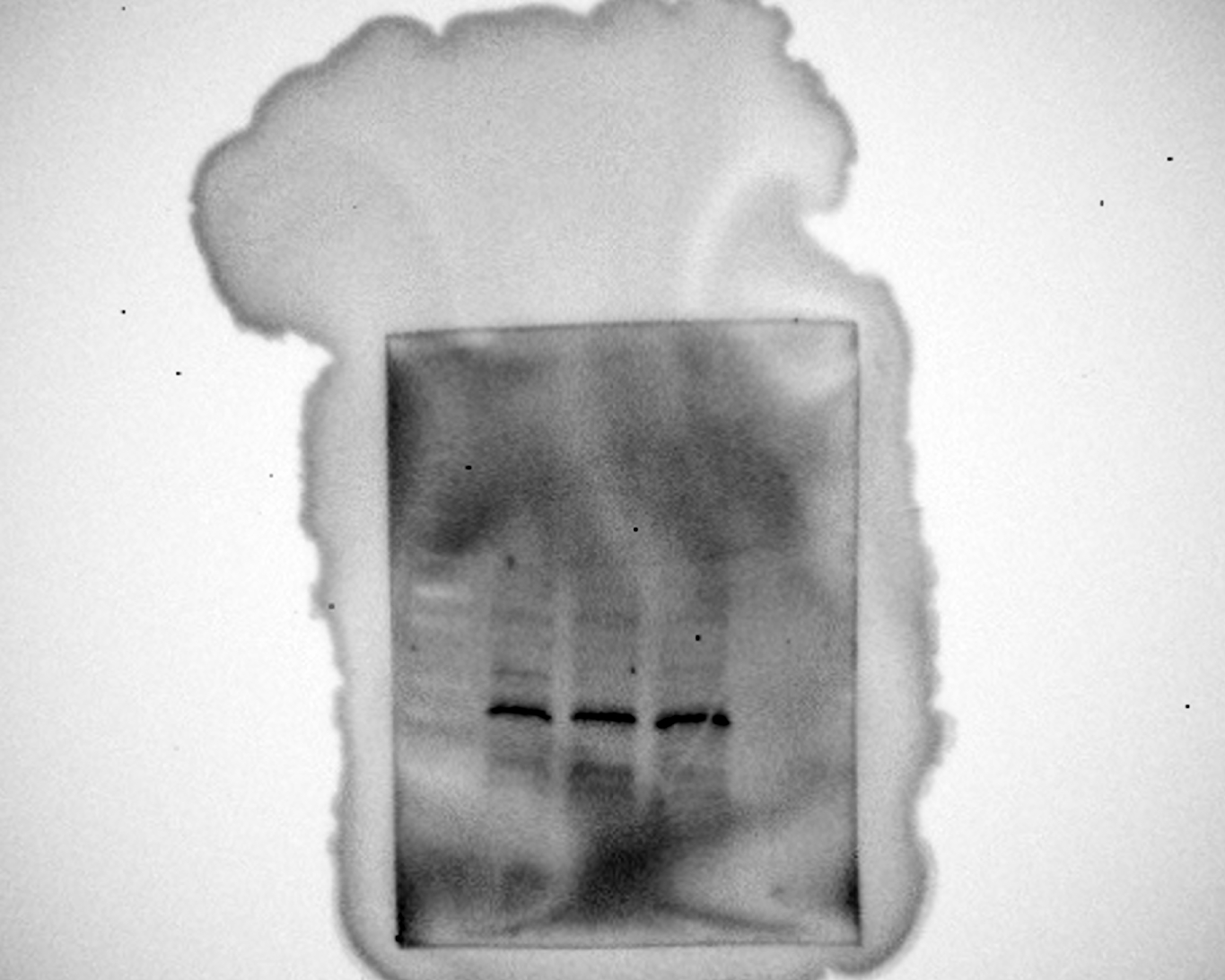

Supplement: Figure 1—figure supplement 1—source data 1. [file elife-78546-fig1-figsupp1-data1.zip › Figure 1 - Suppl fig 1 - Source files/Fig 1 - supp fig 1 - source - ALAD_TUBa.jpg]

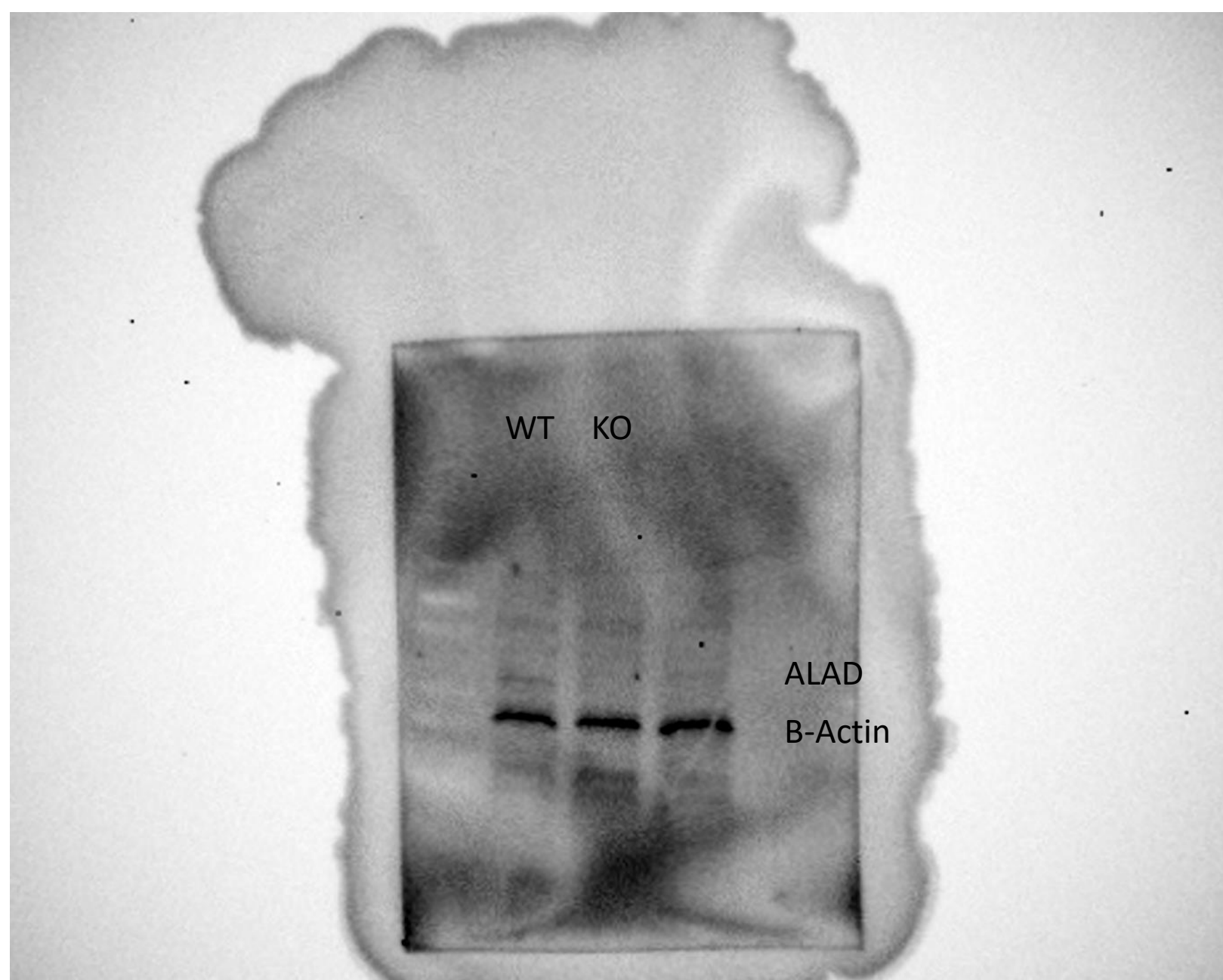

Supplement: Figure 1—figure supplement 1—source data 1. [file elife-78546-fig1-figsupp1-data1.zip › Figure 1 - Suppl fig 1 - Source files/Figure 1 - supplementary figure 1 - source data.pdf]

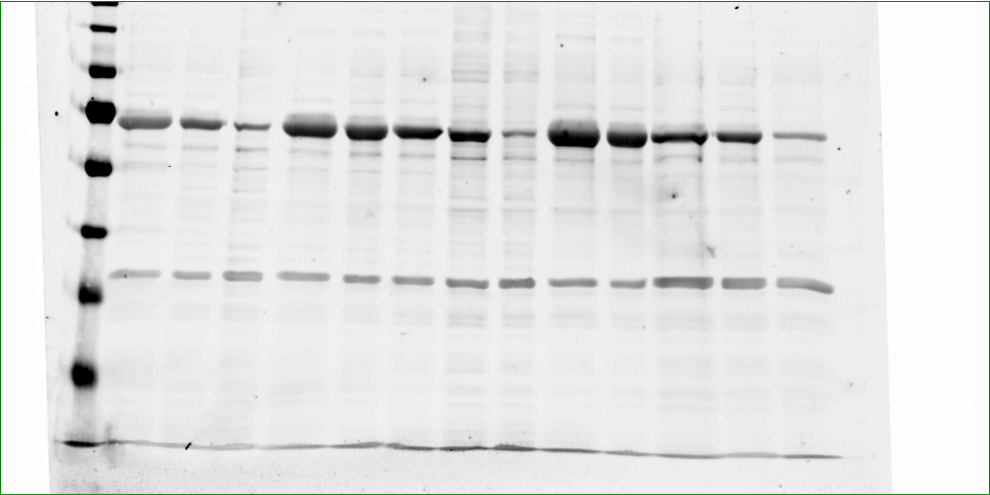

Supplement: Figure 2—source data 1. [file elife-78546-fig2-data1.zip › Figure 2 - Source files 1/Fig 2 - source data 1 - GAPDH.png]

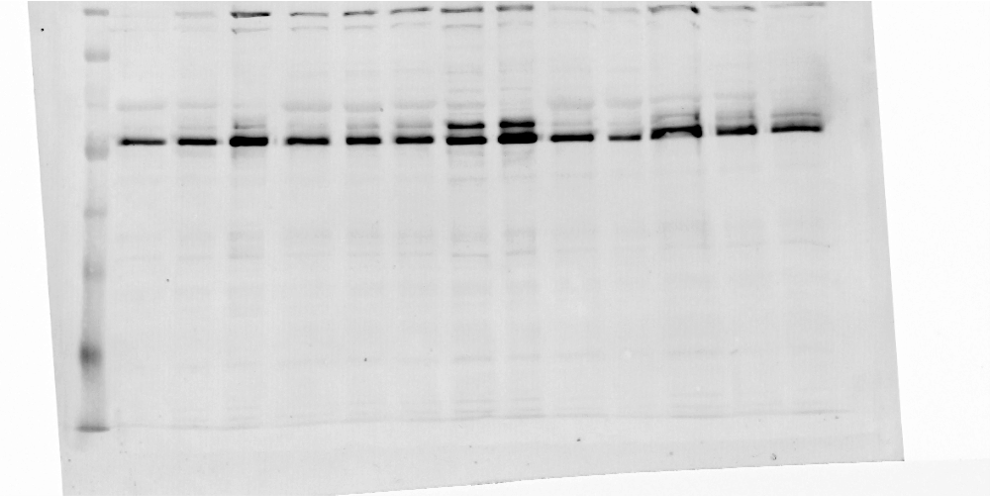

Supplement: Figure 2—source data 1. [file elife-78546-fig2-data1.zip › Figure 2 - Source files 1/Fig 2 - source data 1 - pSMAD3.png]

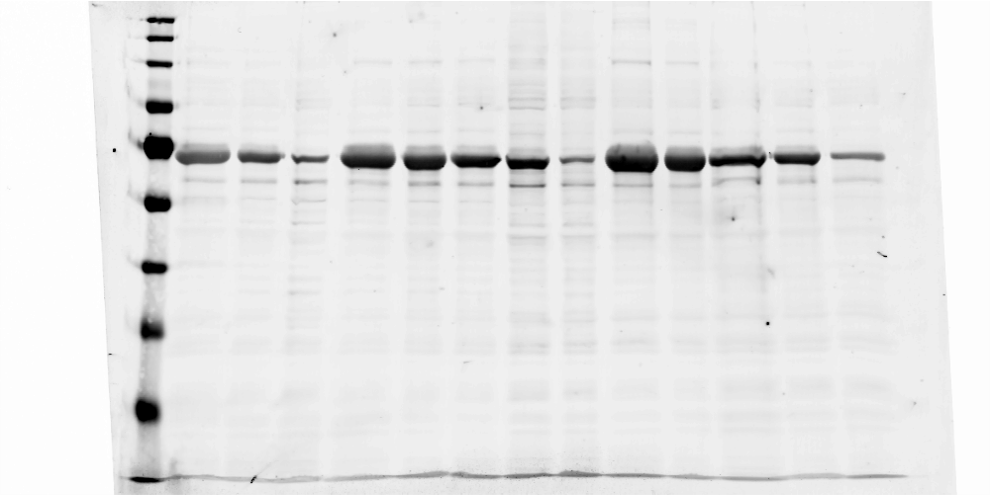

Supplement: Figure 2—source data 1. [file elife-78546-fig2-data1.zip › Figure 2 - Source files 1/Fig 2 - source data 1 - SMAD3.png]

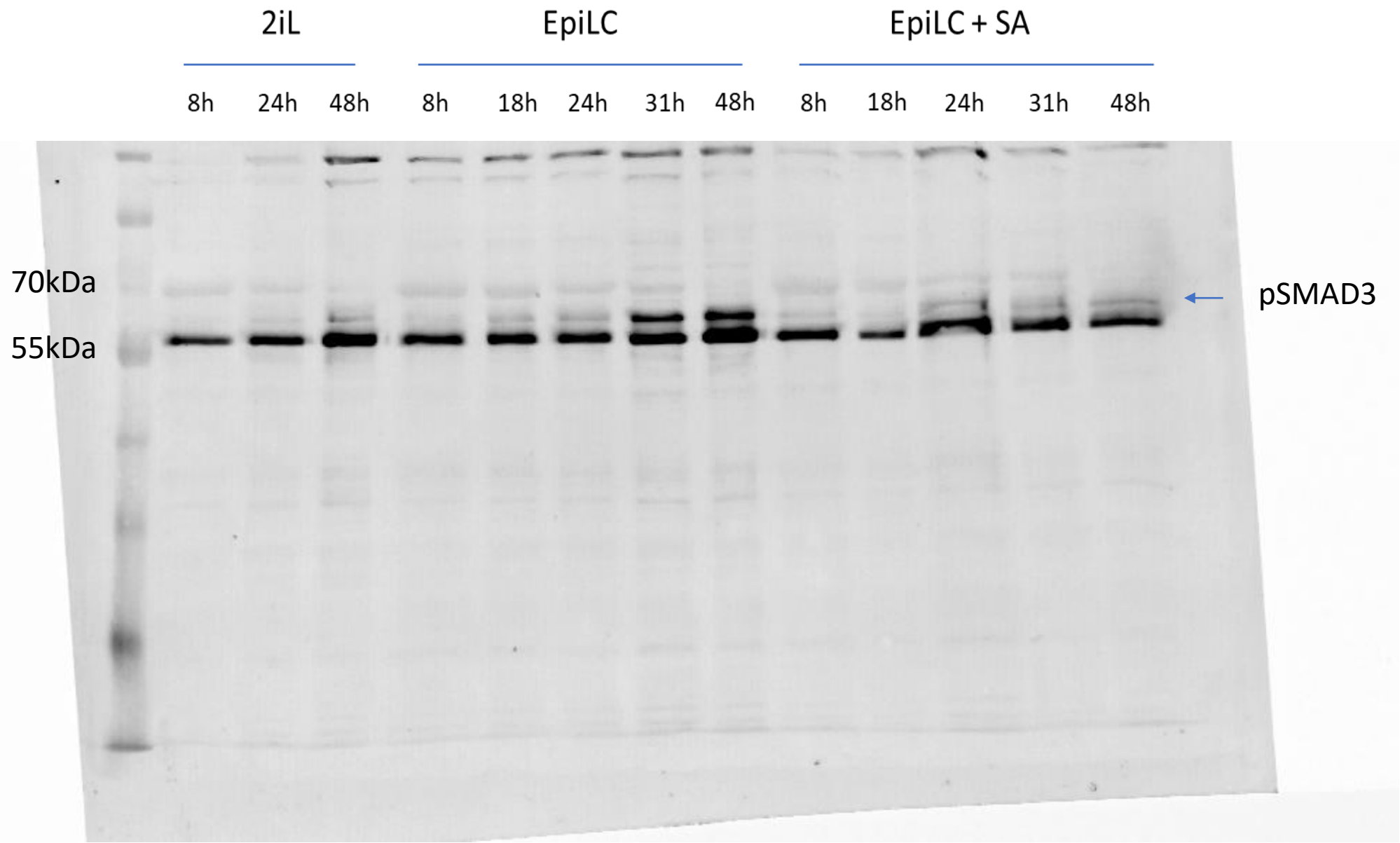

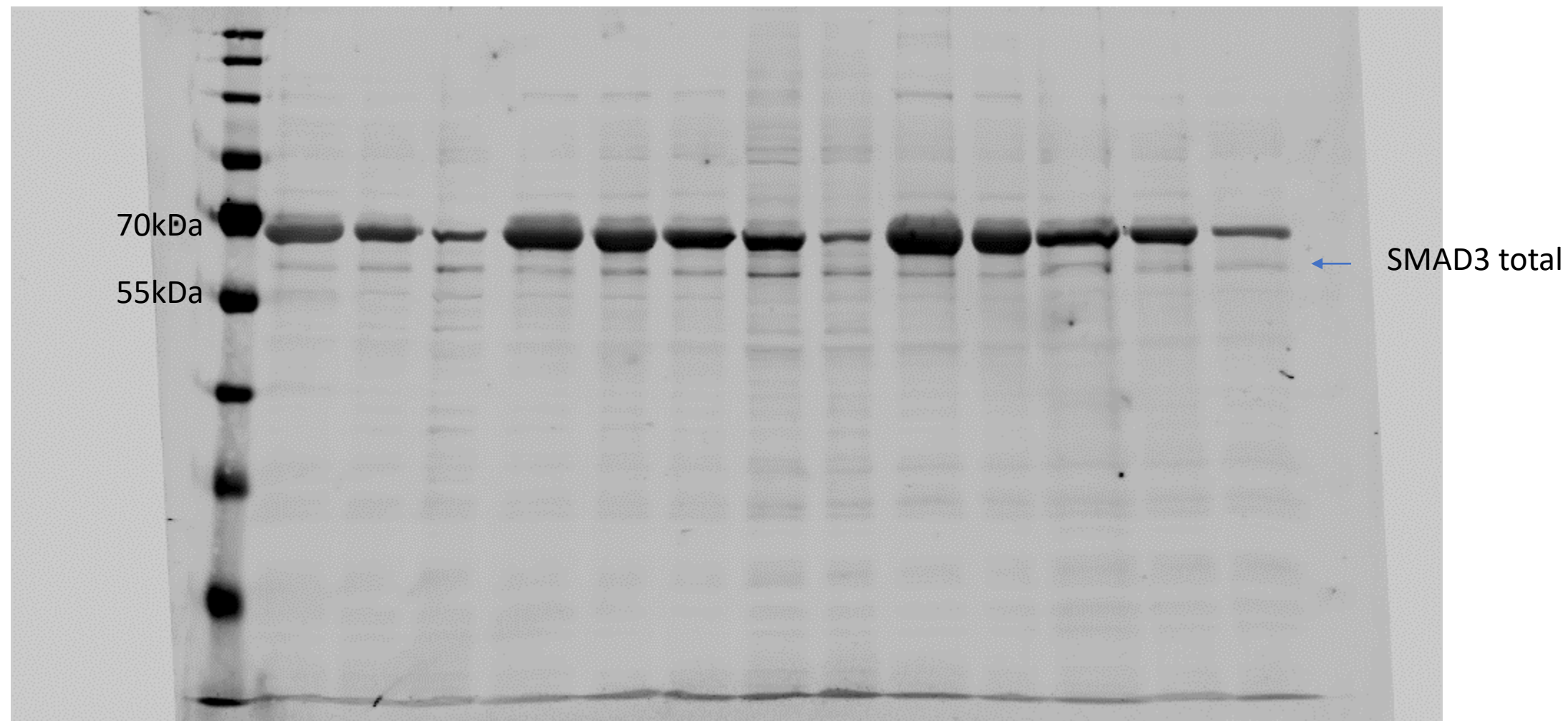

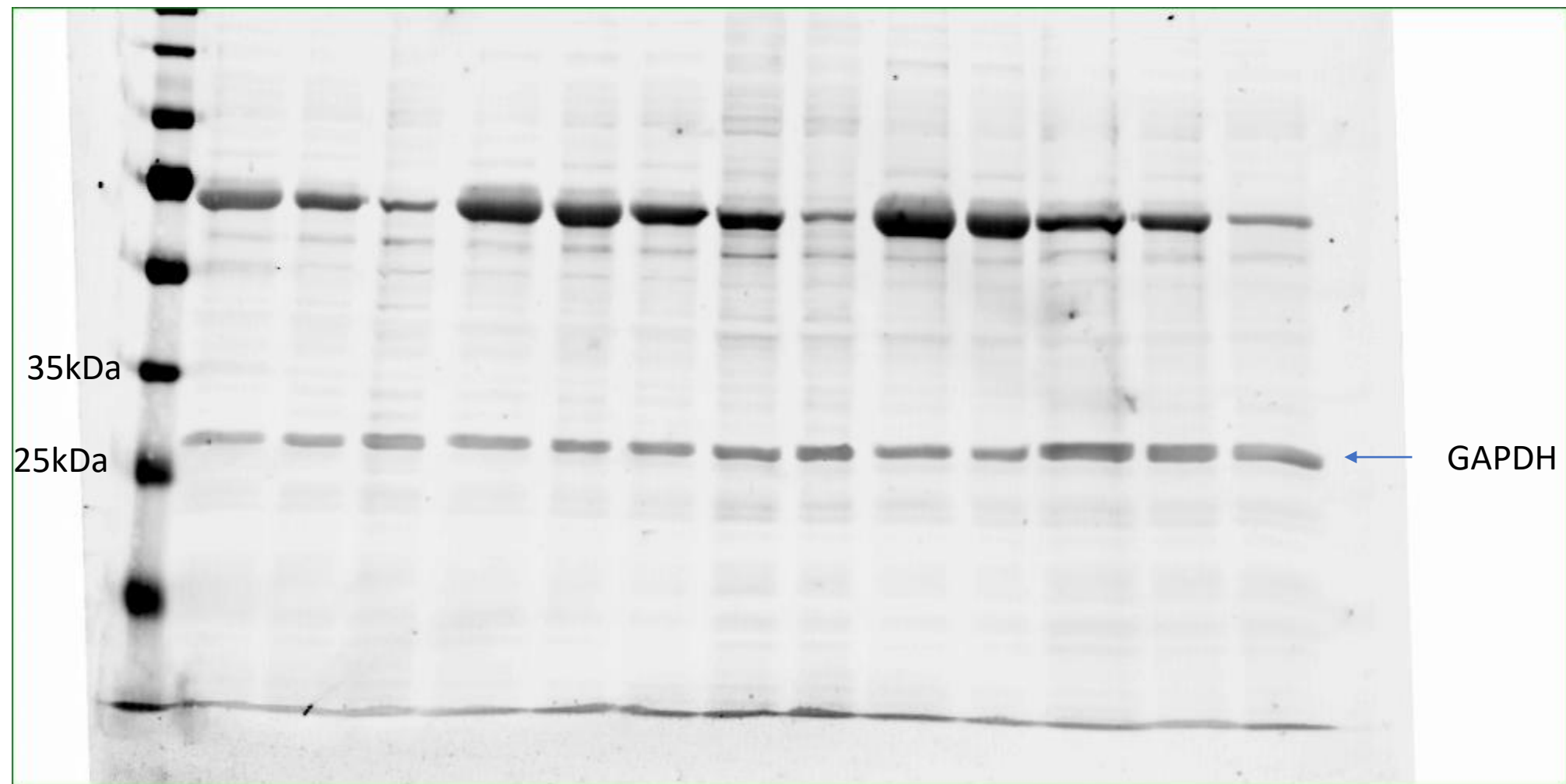

Supplement: Figure 2—source data 1. [file elife-78546-fig2-data1.zip › Figure 2 - Source files 1/figure 2 - source data 1.pdf]

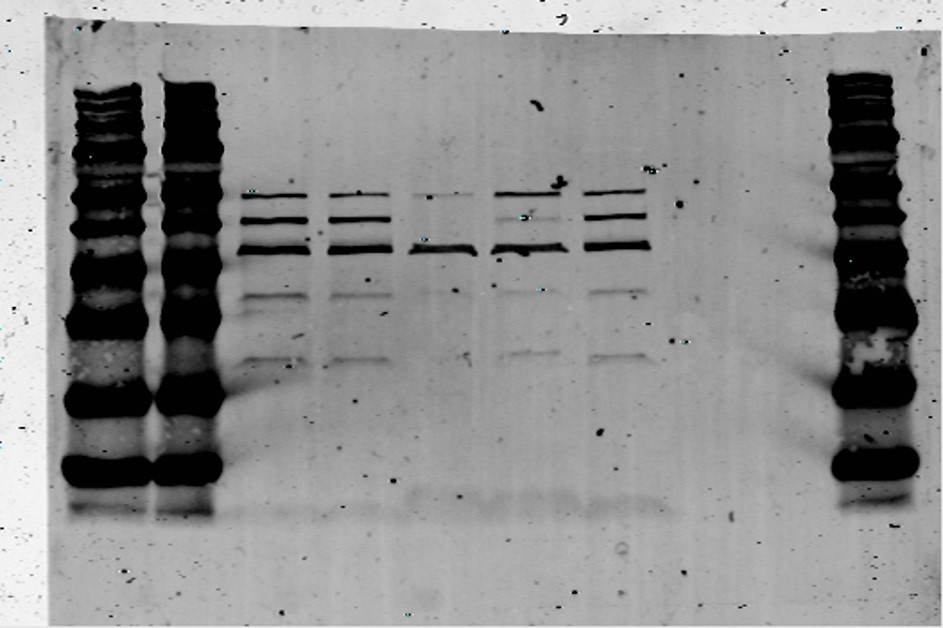

Supplement: Figure 2—source data 2. [file elife-78546-fig2-data2.zip › Figure 2 - Source files 2/Fig 2 - source data 2 - GAPDH.jpg]

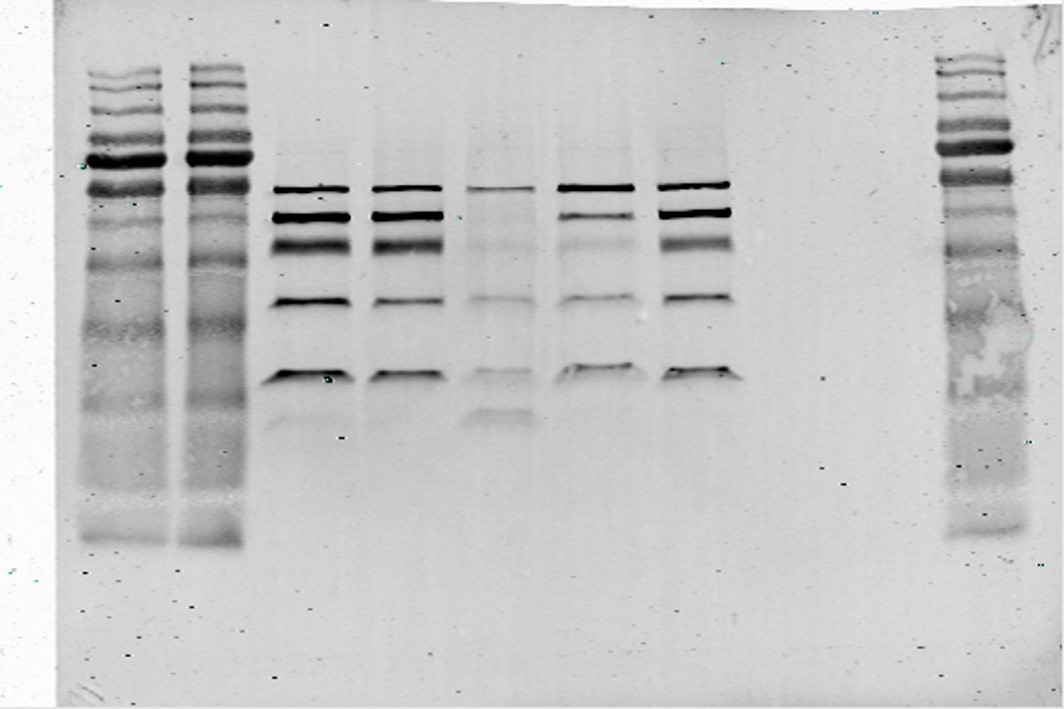

Supplement: Figure 2—source data 2. [file elife-78546-fig2-data2.zip › Figure 2 - Source files 2/Fig 2 - source data 2 - OXPHOS.jpg]

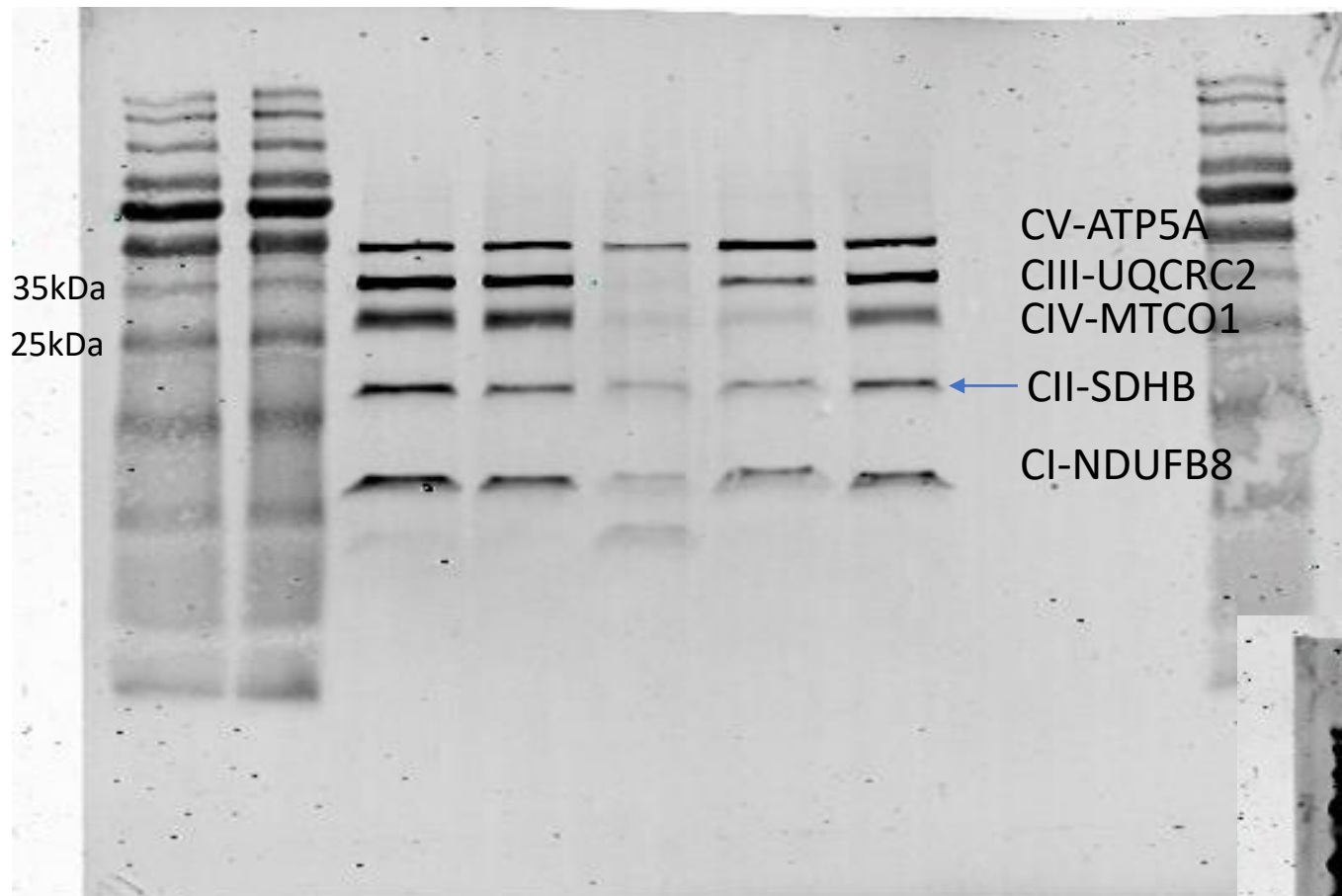

|       | 2iL | EpiLC |   |   |
|-------|-----|-------|---|---|
| SA    | -   | +     | - | + |
| Hemin | -   | -     | - | + |

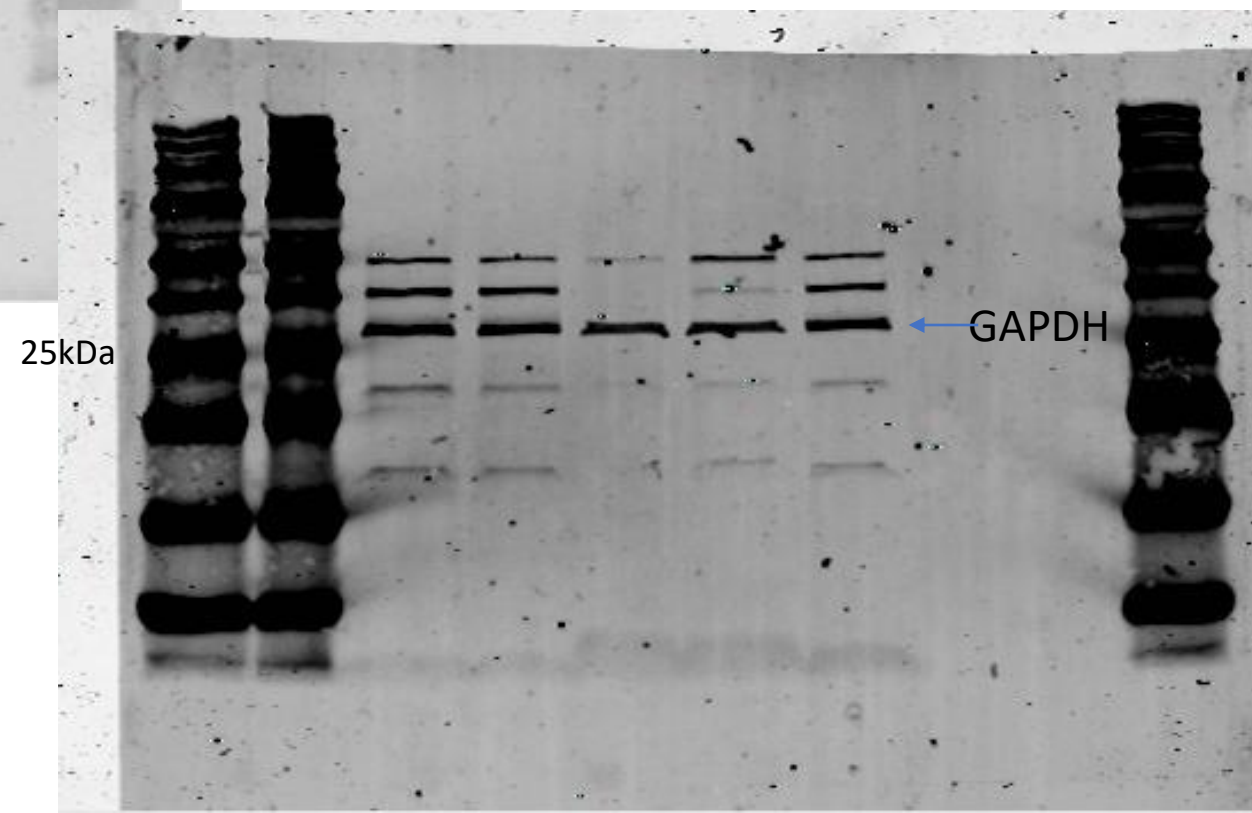

Supplement: Figure 2—source data 2. [file elife-78546-fig2-data2.zip › Figure 2 - Source files 2/figure 2 - source data 2.pdf]

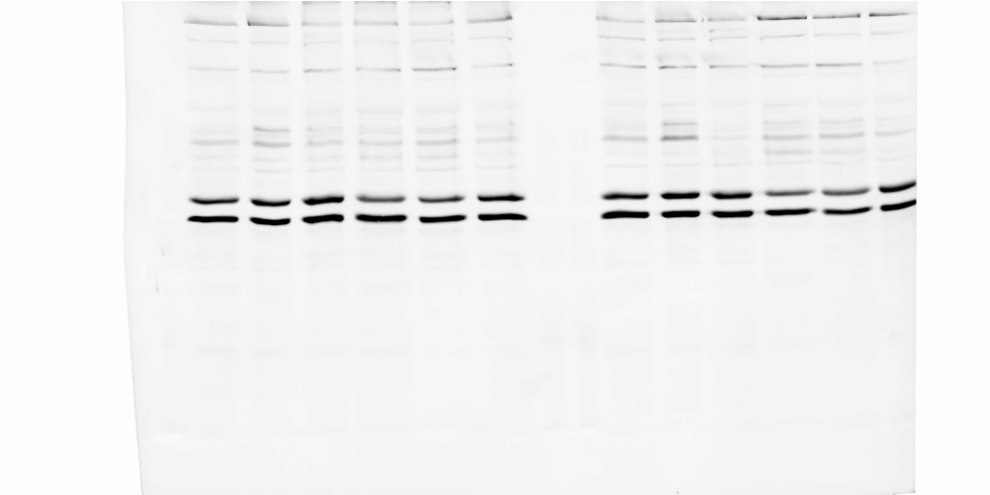

Supplement: Figure 2—source data 3. [file elife-78546-fig2-data3.zip › Figure 2 - Source files 3/figure 2- source data 3 - ERK.png]

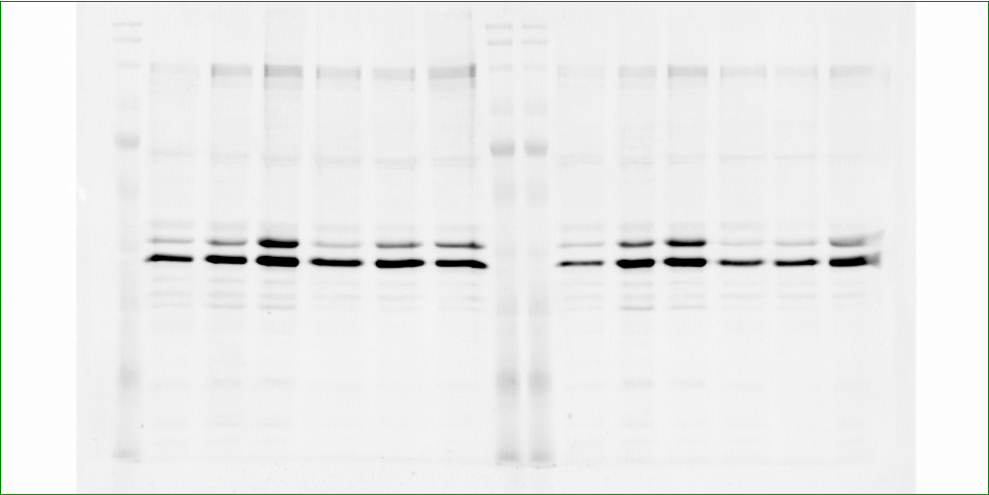

Supplement: Figure 2—source data 3. [file elife-78546-fig2-data3.zip › Figure 2 - Source files 3/figure 2- source data 3 - pERK.png]

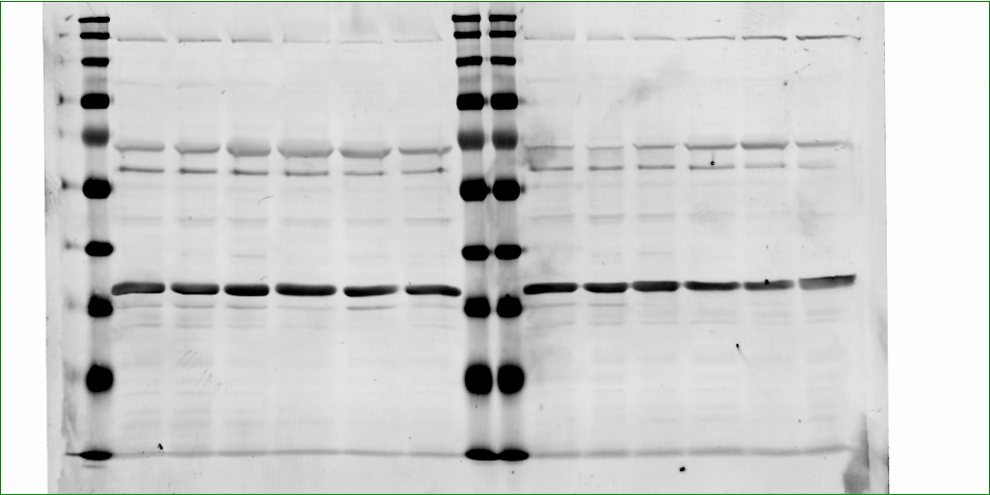

Supplement: Figure 2—source data 3. [file elife-78546-fig2-data3.zip › Figure 2 - Source files 3/figure 2- source data 3 - SMAD3.png]

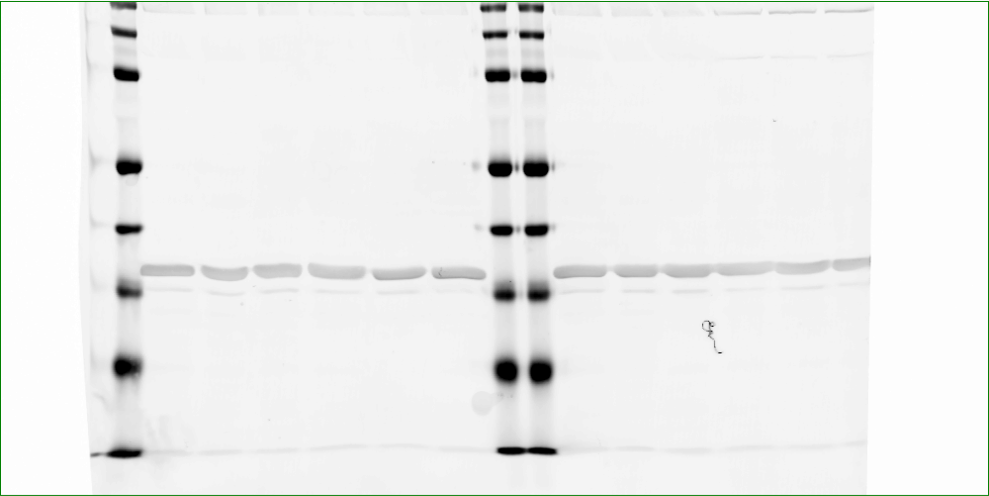

Supplement: Figure 2—source data 3. [file elife-78546-fig2-data3.zip › Figure 2 - Source files 3/figure 2- source data 3 GAPDH.png]

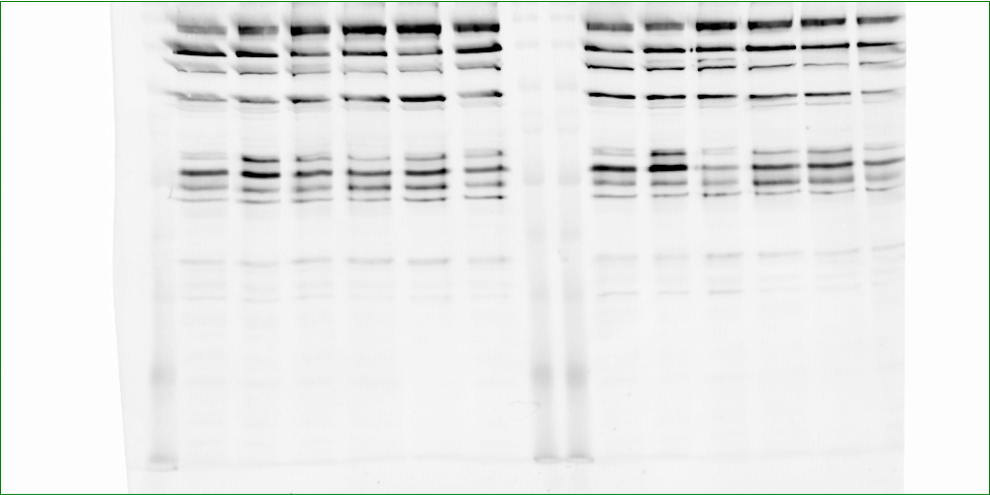

Supplement: Figure 2—source data 3. [file elife-78546-fig2-data3.zip › Figure 2 - Source files 3/figure 2- source data 3 pSMAD3.png]

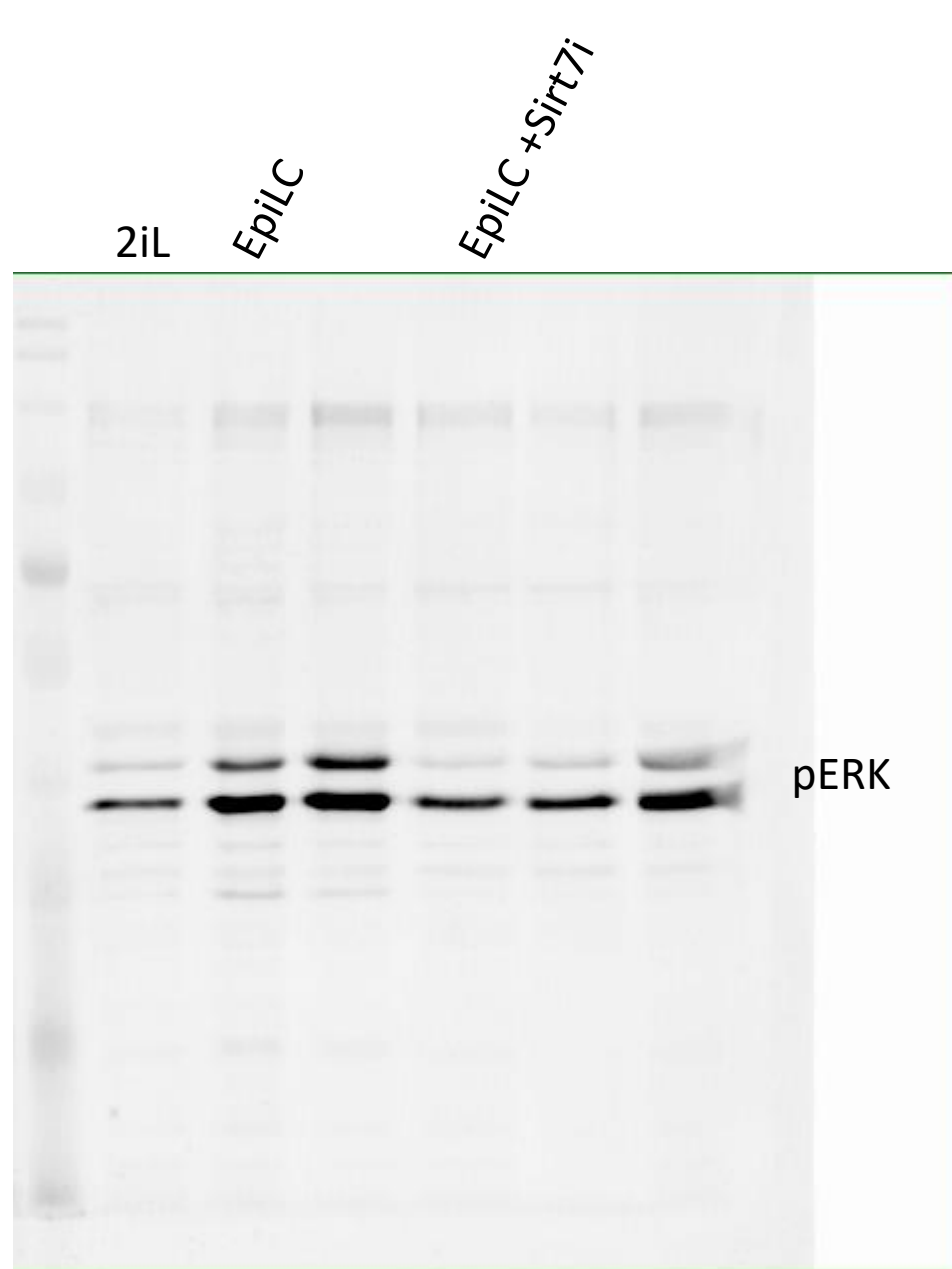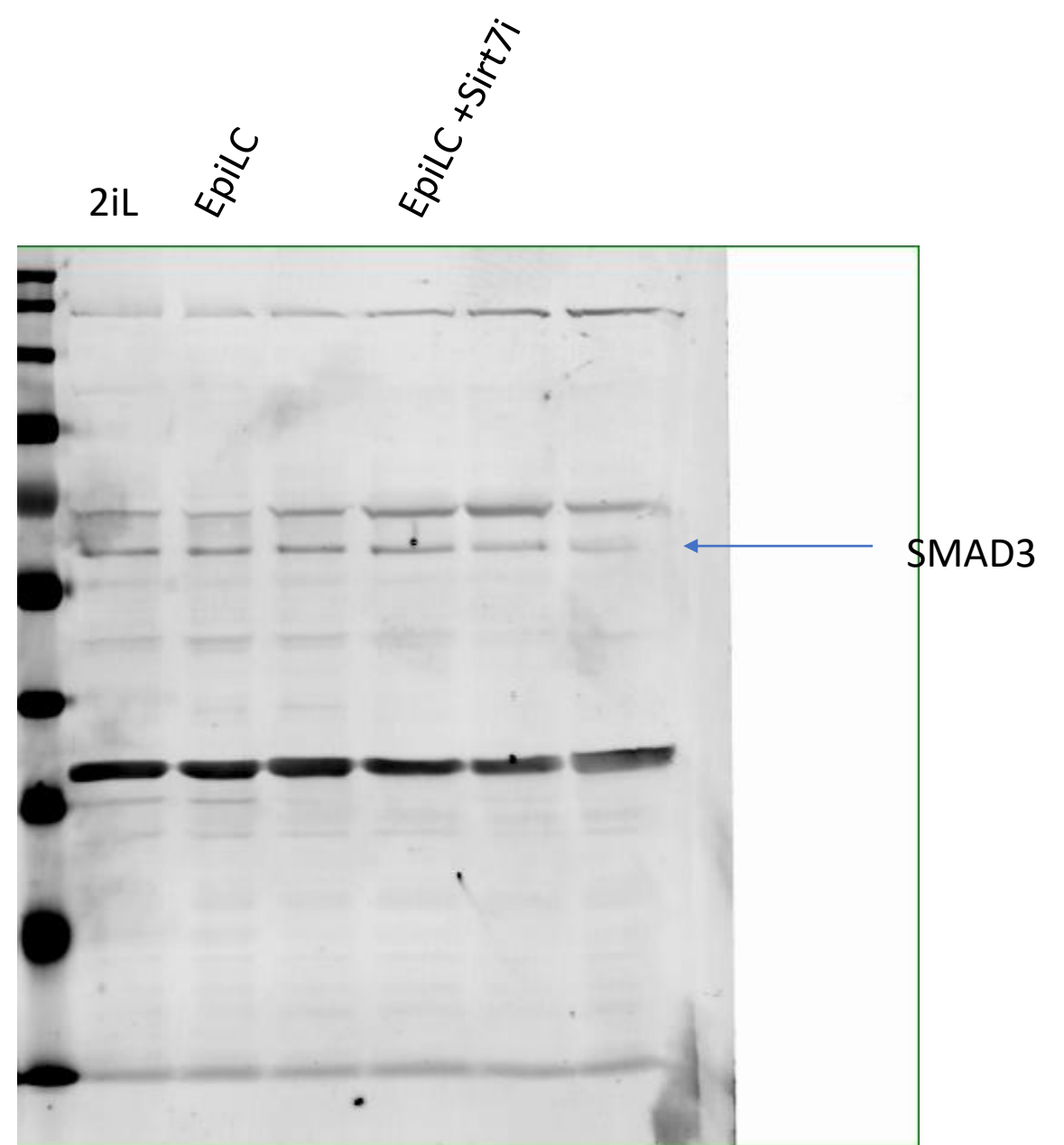

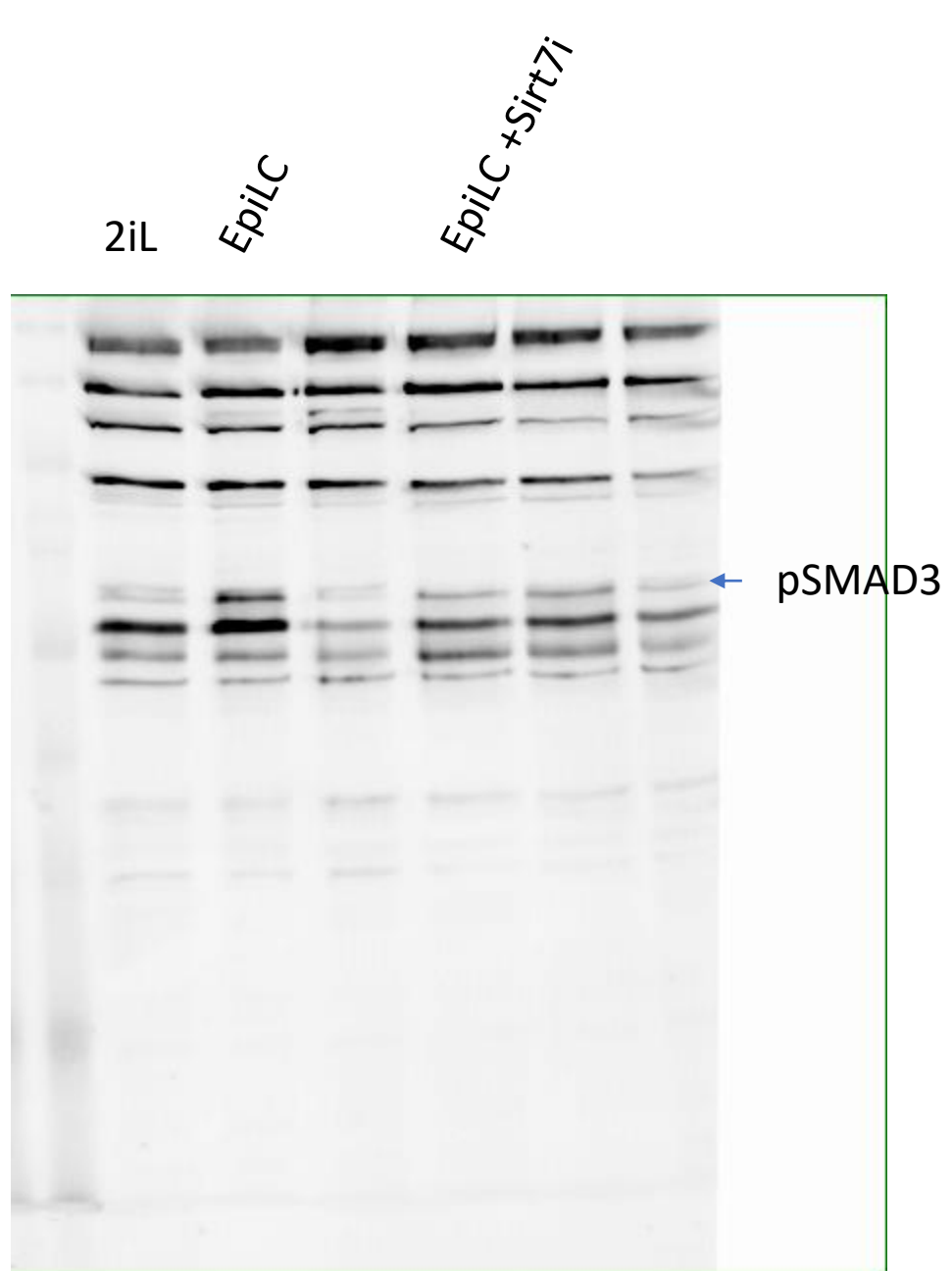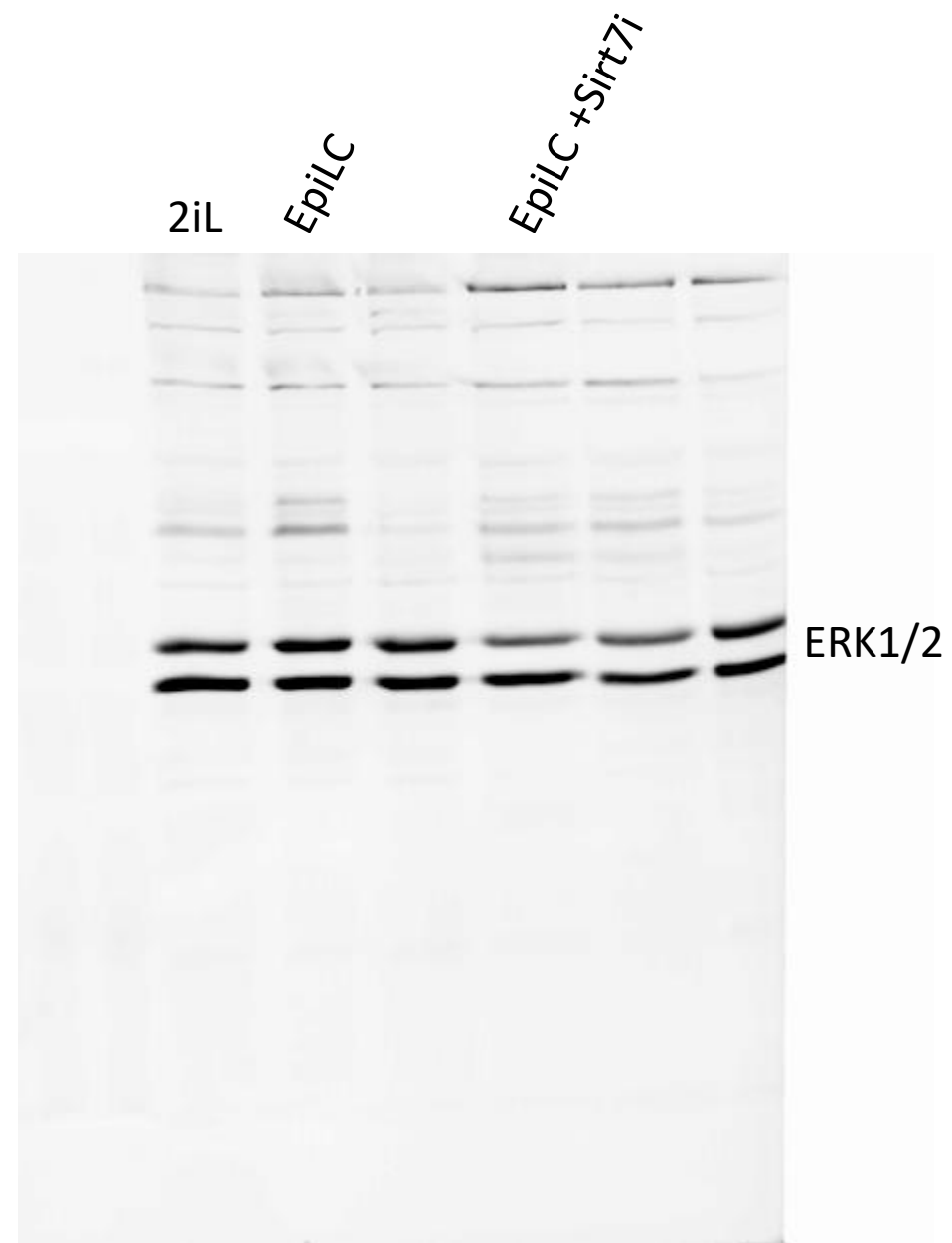

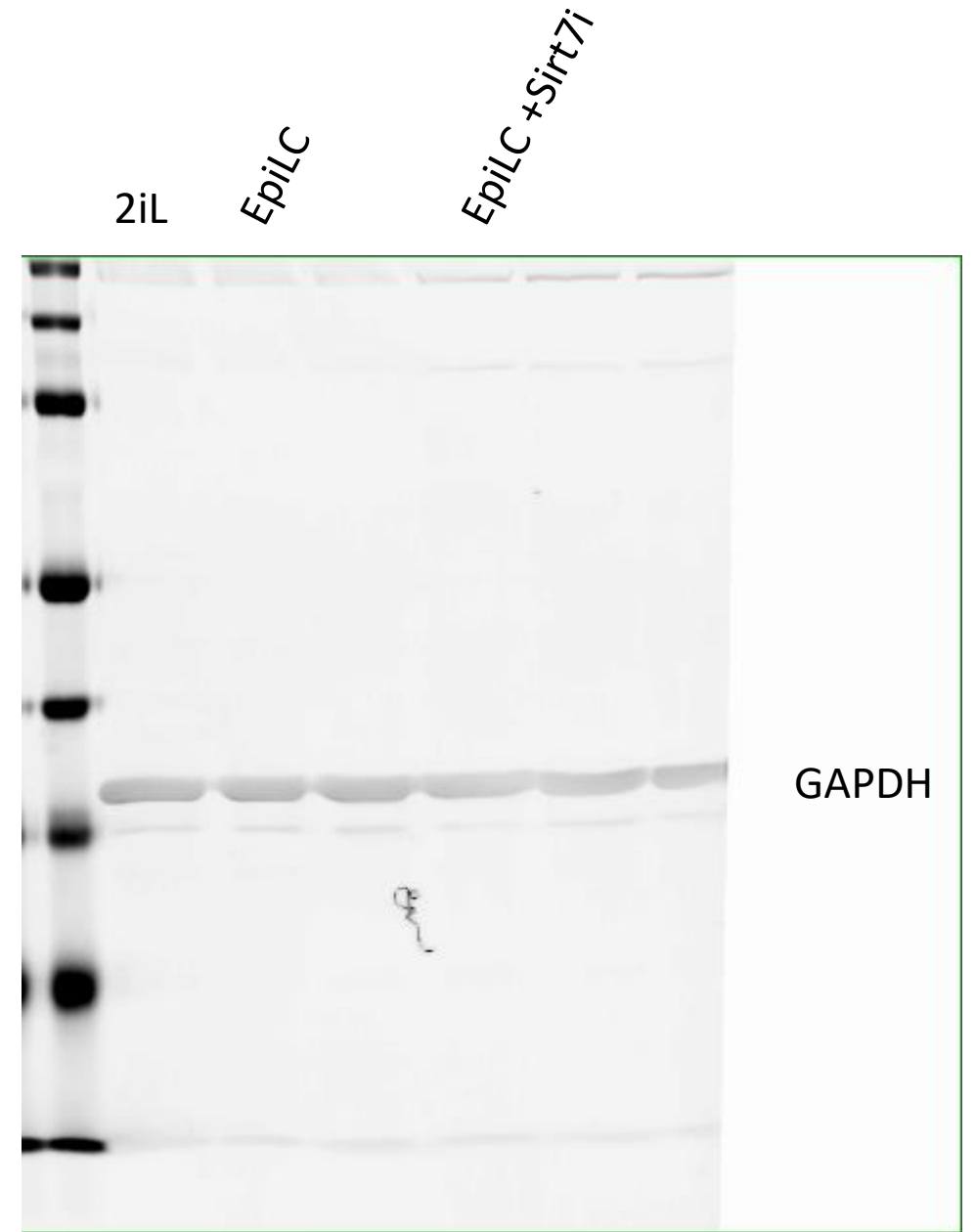

Supplement: Figure 2—source data 3. [file elife-78546-fig2-data3.zip › Figure 2 - Source files 3/figure 2- source data 3.pdf]

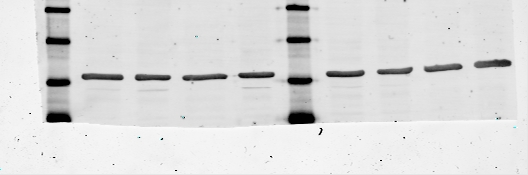

Supplement: Figure 2—figure supplement 1—source data 1. [file elife-78546-fig2-figsupp1-data1.zip › Figure 2 - Suppl fig 1 - Source files 1/Figure 2 - suppl fig 1 - source files 1 - GAPDH.jpg]

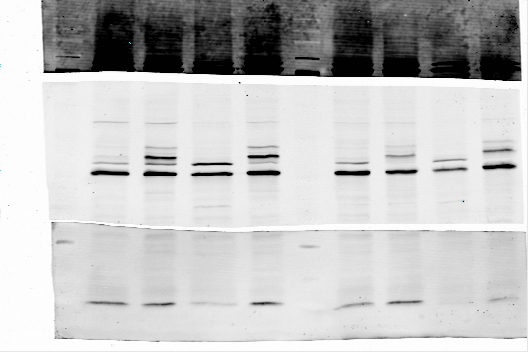

Supplement: Figure 2—figure supplement 1—source data 1. [file elife-78546-fig2-figsupp1-data1.zip › Figure 2 - Suppl fig 1 - Source files 1/Figure 2 - suppl fig 1 - source files 1 - pEIF2a.jpg]

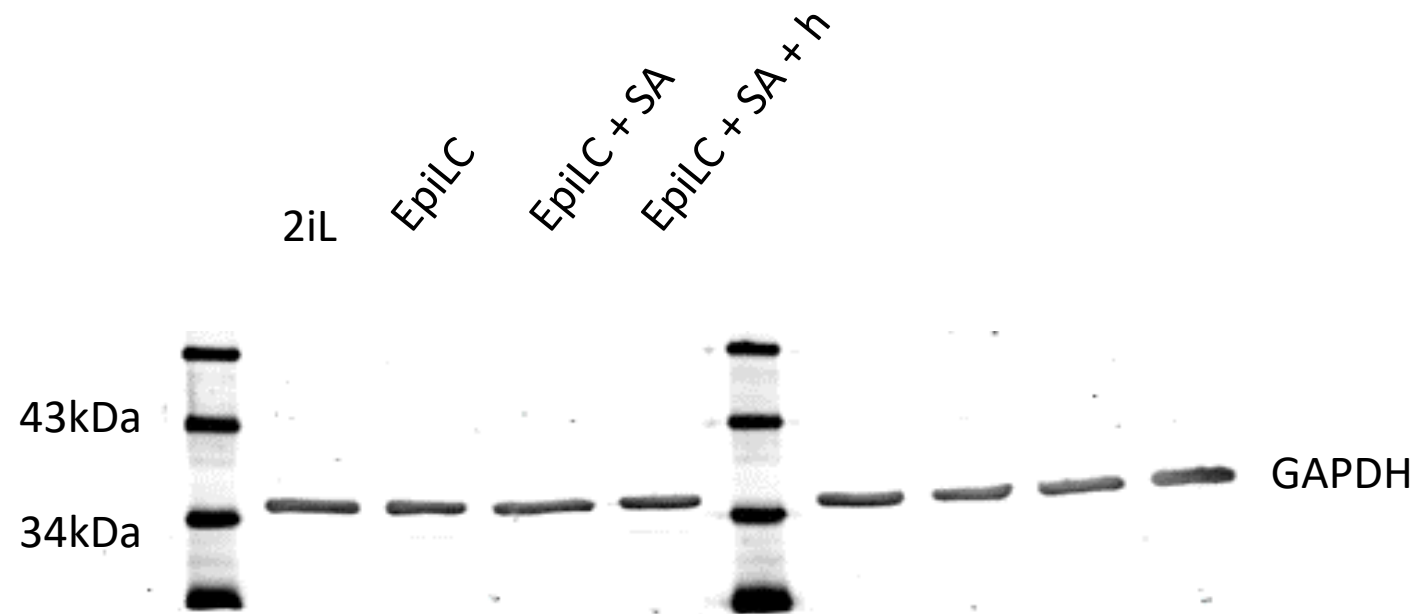

2iL      EpilC      EpilC + SA      EpilC + SA + h

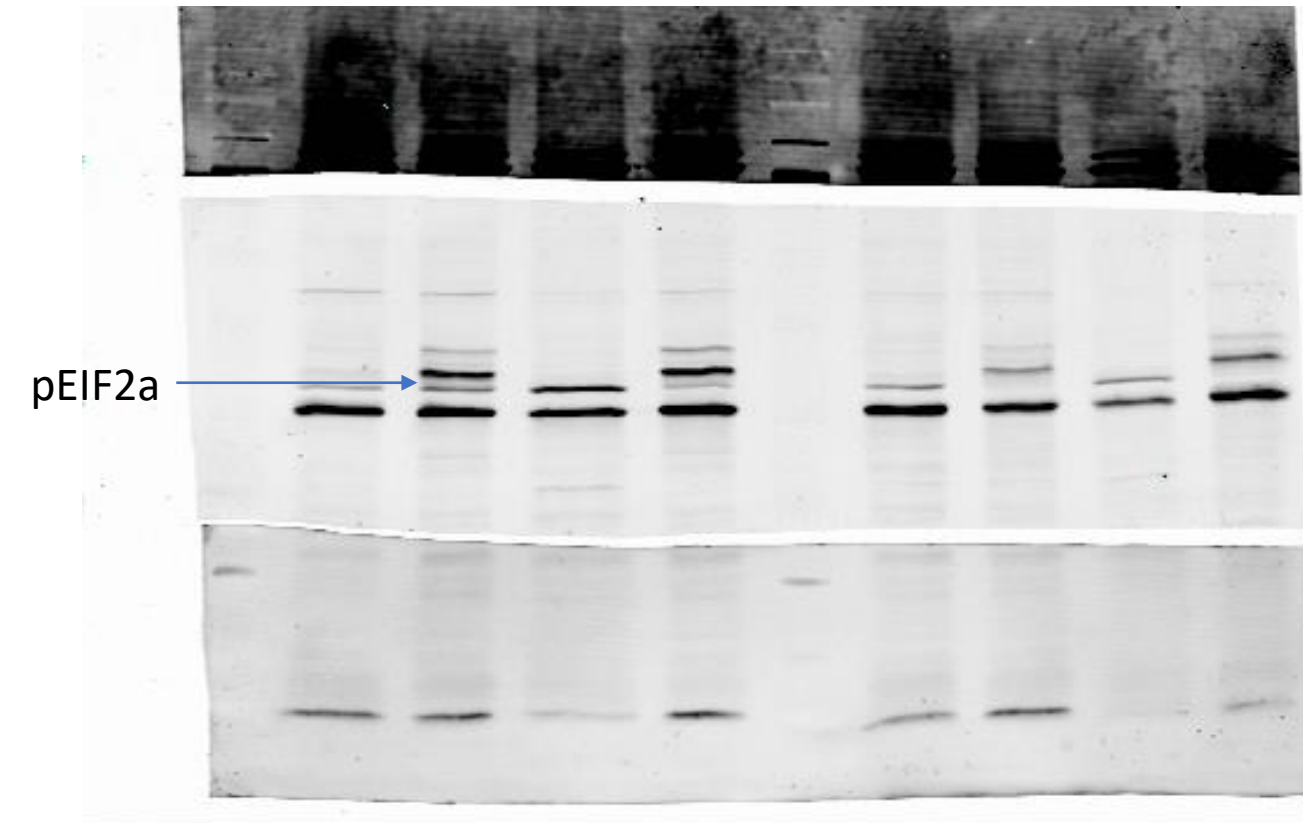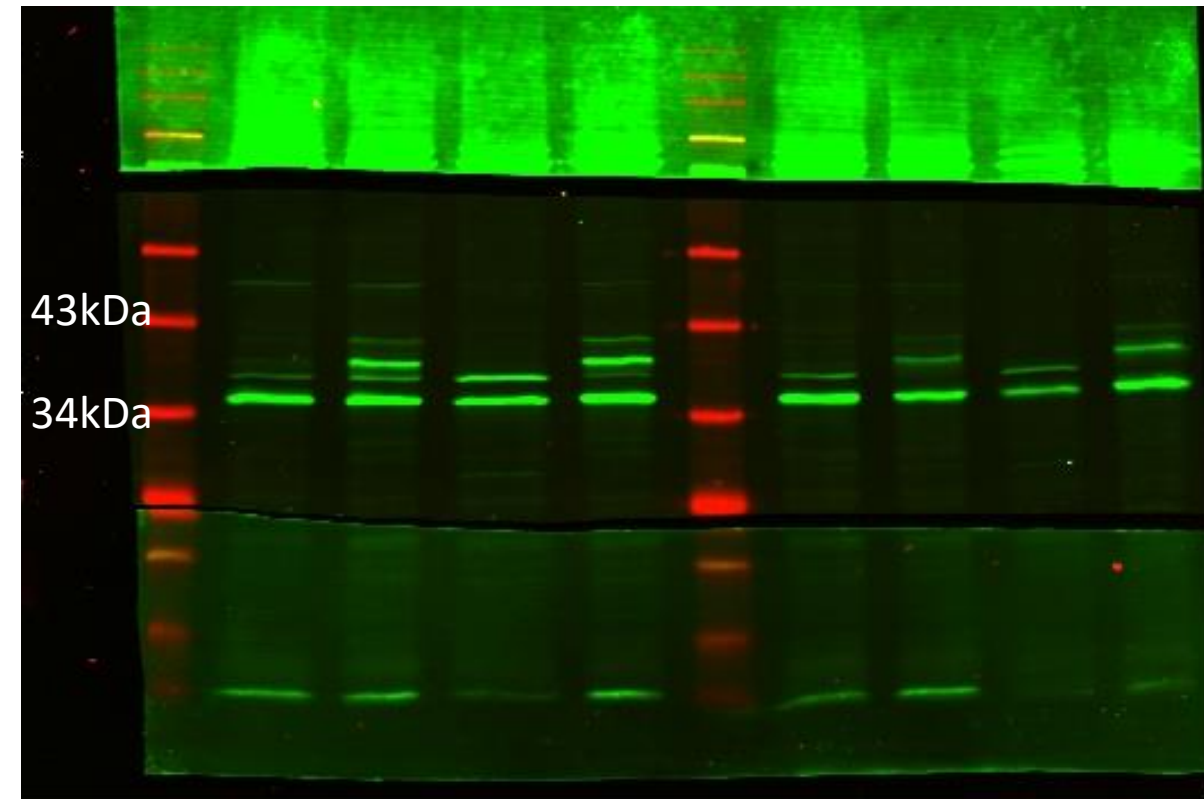

Supplement: Figure 2—figure supplement 1—source data 1. [file elife-78546-fig2-figsupp1-data1.zip › Figure 2 - Suppl fig 1 - Source files 1/Figure 2 - suppl fig 1 - source files 1.pdf]

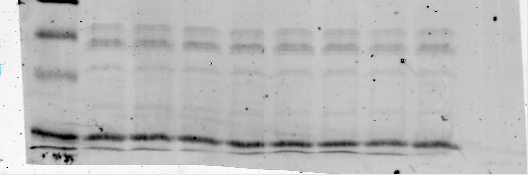

Supplement: Figure 2—figure supplement 1—source data 2. [file elife-78546-fig2-figsupp1-data2.zip › Figure 2 - Suppl fig 1 - Source files 2/Figure 2 - suppl fig 1 - source files 2 - H3.jpg]

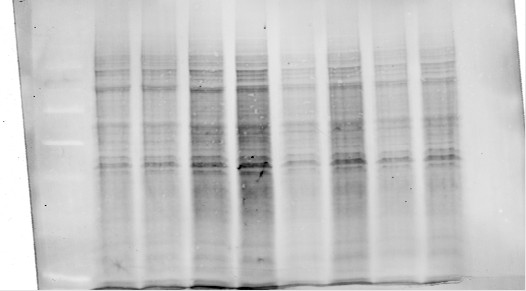

Supplement: Figure 2—figure supplement 1—source data 2. [file elife-78546-fig2-figsupp1-data2.zip › Figure 2 - Suppl fig 1 - Source files 2/Figure 2 - suppl fig 1 - source files 2 - Puro.jpg]

Puromycin-labeled peptides

|        | 2iL |   | EpiLC 48h |   |
|--------|-----|---|-----------|---|
| SA:    | -   | - | +         | + |
| Hemin: | -   | - | -         | + |

180 kDa  
130 kDa  
95 kDa  
72 kDa  
55 kDa  
43 kDa  
34 kDa  
26 kDa

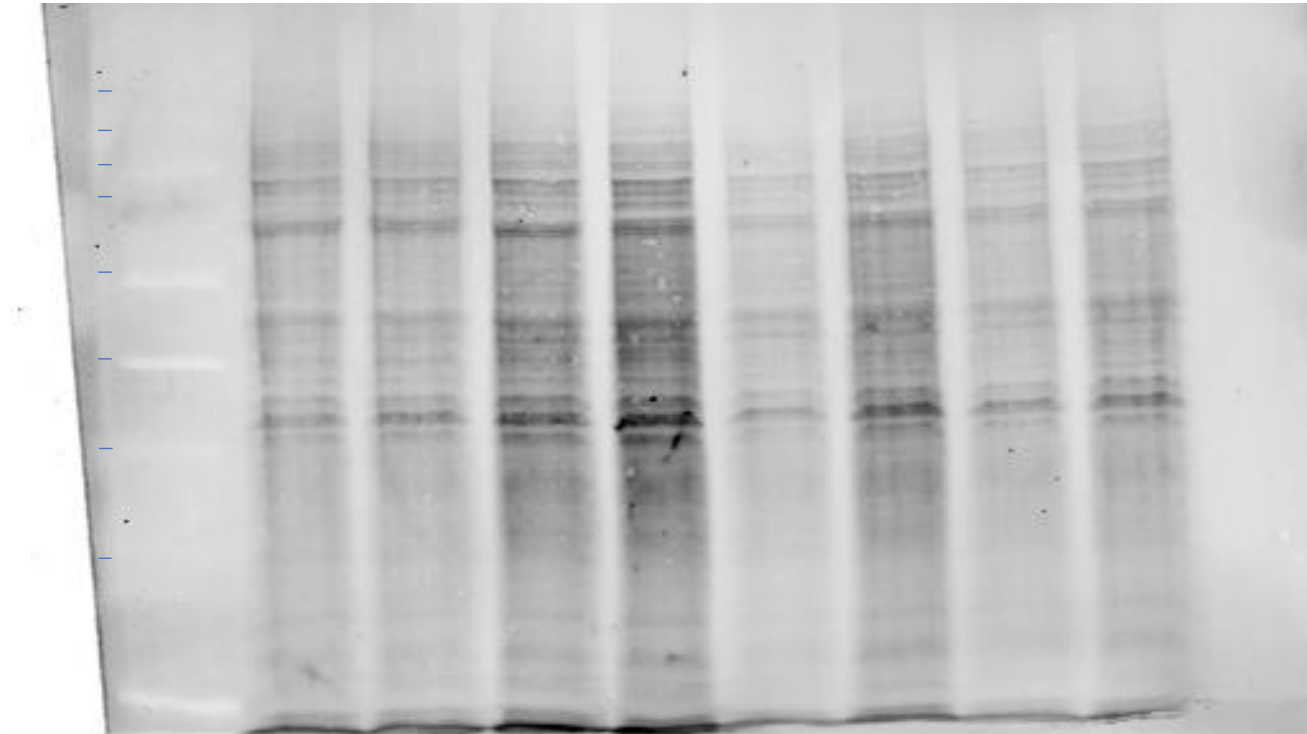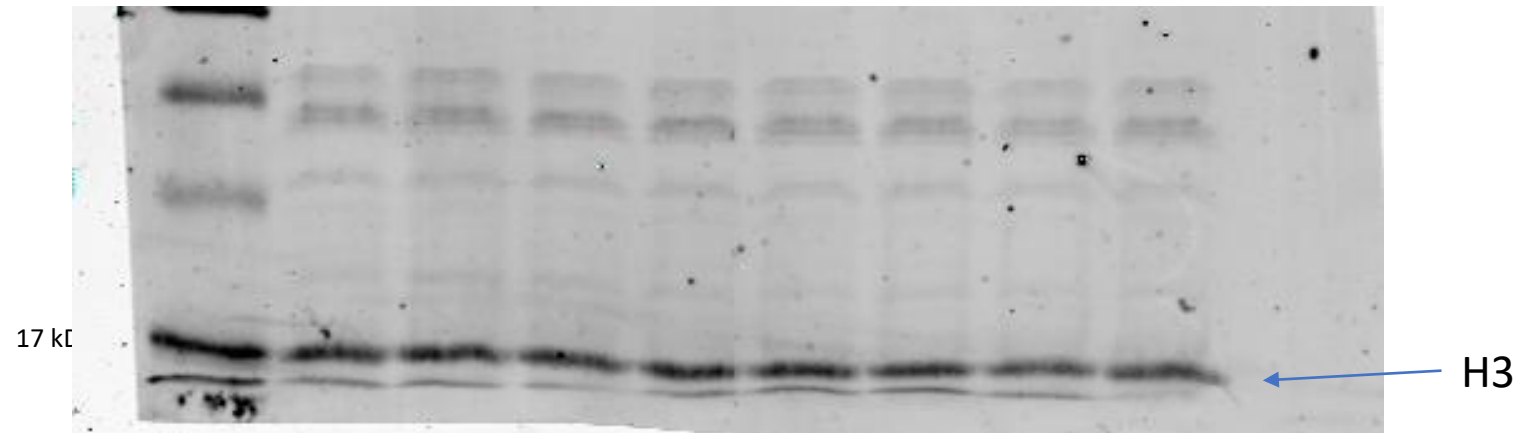

Supplement: Figure 2—figure supplement 1—source data 2. [file elife-78546-fig2-figsupp1-data2.zip › Figure 2 - Suppl fig 1 - Source files 2/Figure 2 - suppl fig 1 - source files 2.pdf]

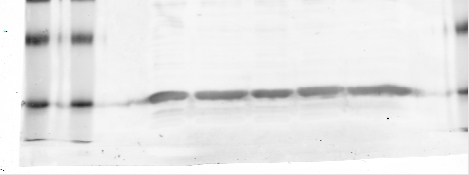

Supplement: Figure 2—figure supplement 1—source data 3. [file elife-78546-fig2-figsupp1-data3.zip › Figure 2 - Suppl fig 1 - Source files 3/Figure 2 - suppl fig 1 - source files 3 - H3.jpg]

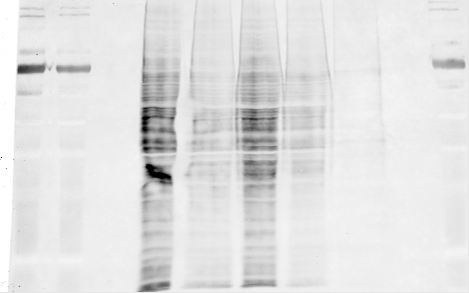

Supplement: Figure 2—figure supplement 1—source data 3. [file elife-78546-fig2-figsupp1-data3.zip › Figure 2 - Suppl fig 1 - Source files 3/Figure 2 - suppl fig 1 - source files 3 - Puro.jpg]

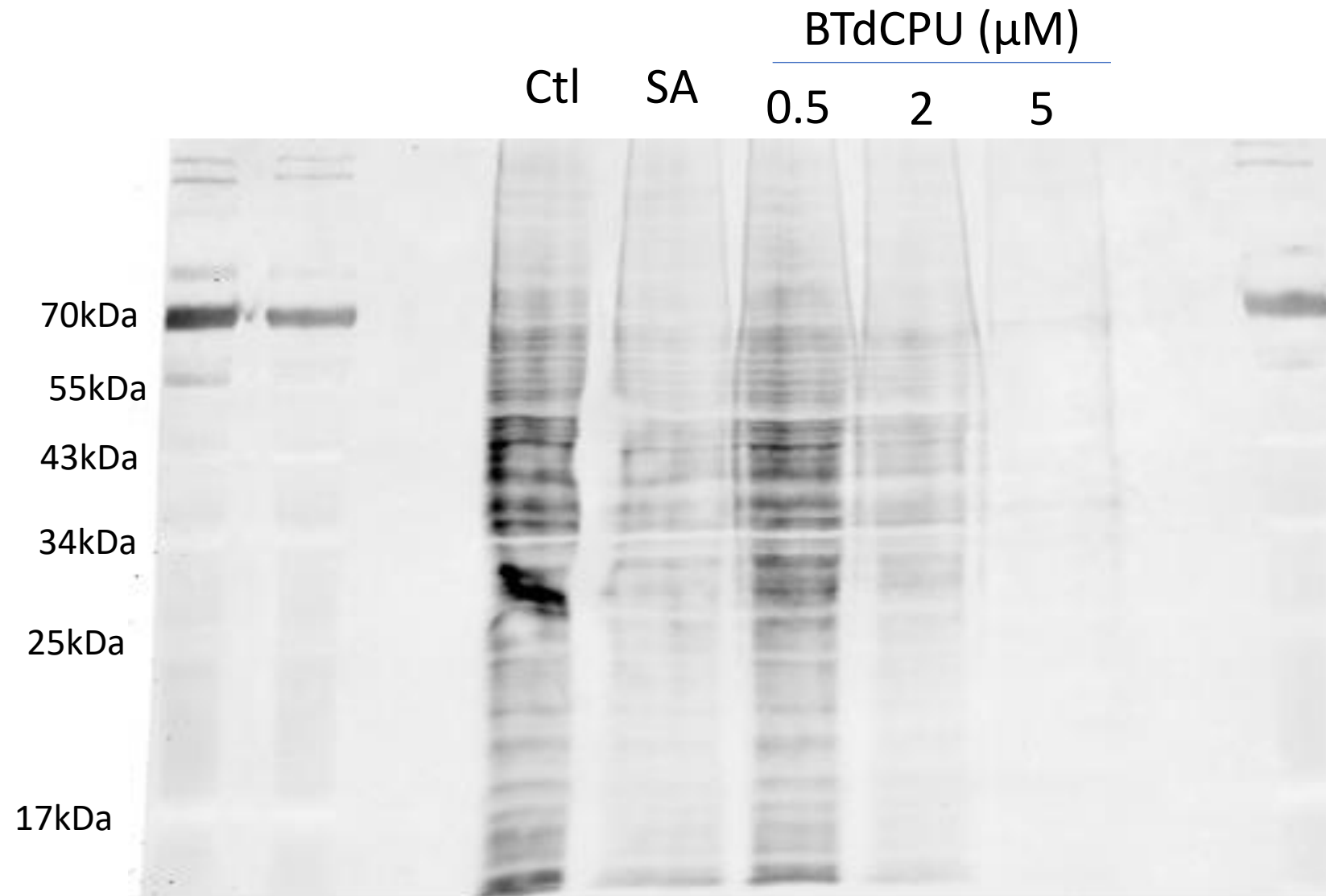

Puromycin-labelled peptides

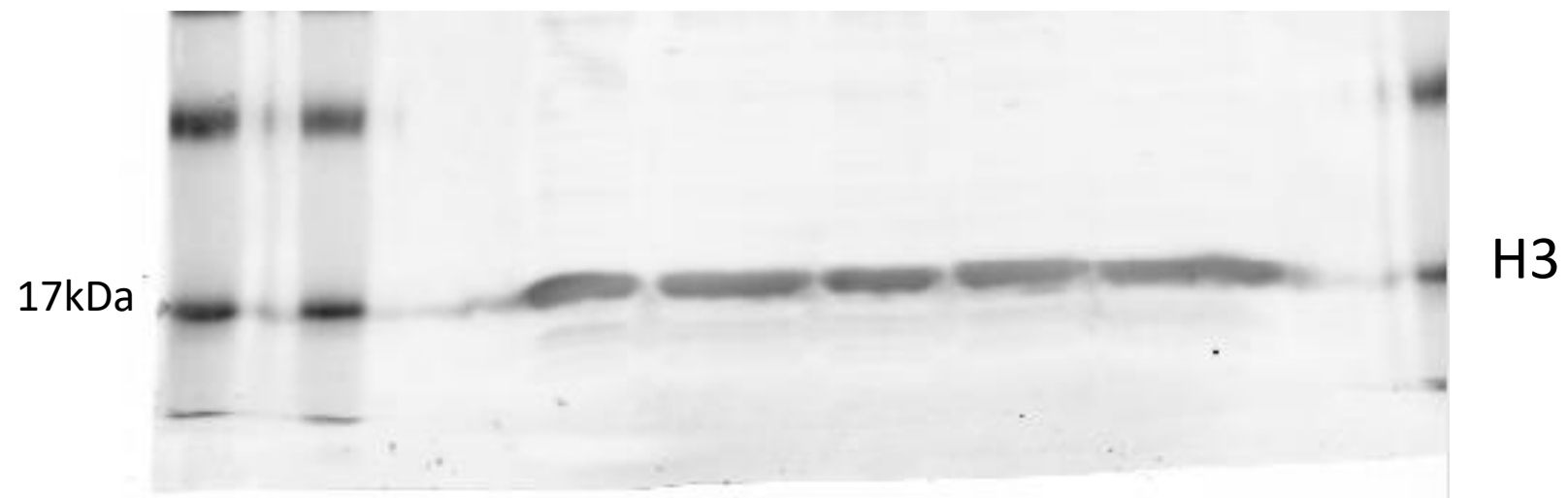

Supplement: Figure 2—figure supplement 1—source data 3. [file elife-78546-fig2-figsupp1-data3.zip › Figure 2 - Suppl fig 1 - Source files 3/Figure 2 - suppl fig 1 - source files 3.pdf]

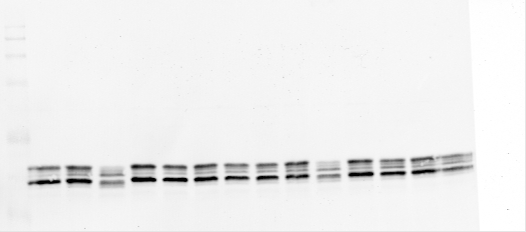

Supplement: Figure 2—figure supplement 1—source data 4. [file elife-78546-fig2-figsupp1-data4.zip › Figure 2 - Suppl fig 1 - Source files 4/Figure 2 supplementary figure 1 - source files 4 - ERK.jpg]

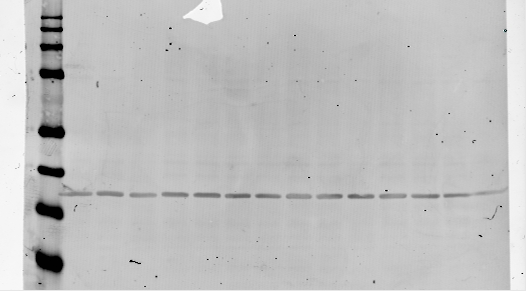

Supplement: Figure 2—figure supplement 1—source data 4. [file elife-78546-fig2-figsupp1-data4.zip › Figure 2 - Suppl fig 1 - Source files 4/Figure 2 supplementary figure 1 - source files 4 - GAPDH.jpg]

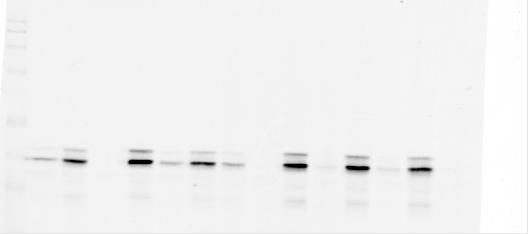

Supplement: Figure 2—figure supplement 1—source data 4. [file elife-78546-fig2-figsupp1-data4.zip › Figure 2 - Suppl fig 1 - Source files 4/Figure 2 supplementary figure 1 - source files 4 - pERK.jpg]
