# Supplementary material for: A critical role for heme synthesis and succinate in the regulation of pluripotent states transitions
Source: eLife. 2023 Jul 10;12:e78546. doi: 10.7554/eLife.78546 (PMC10425175; doi:10.7554/eLife.78546)
Supplement: Figure 2—figure supplement 1—source data 4. [file elife-78546-fig2-figsupp1-data4.zip › Figure 2 - Suppl fig 1 - Source files 4/Figure 2 supplementary figure 1 - source files 4.pdf]

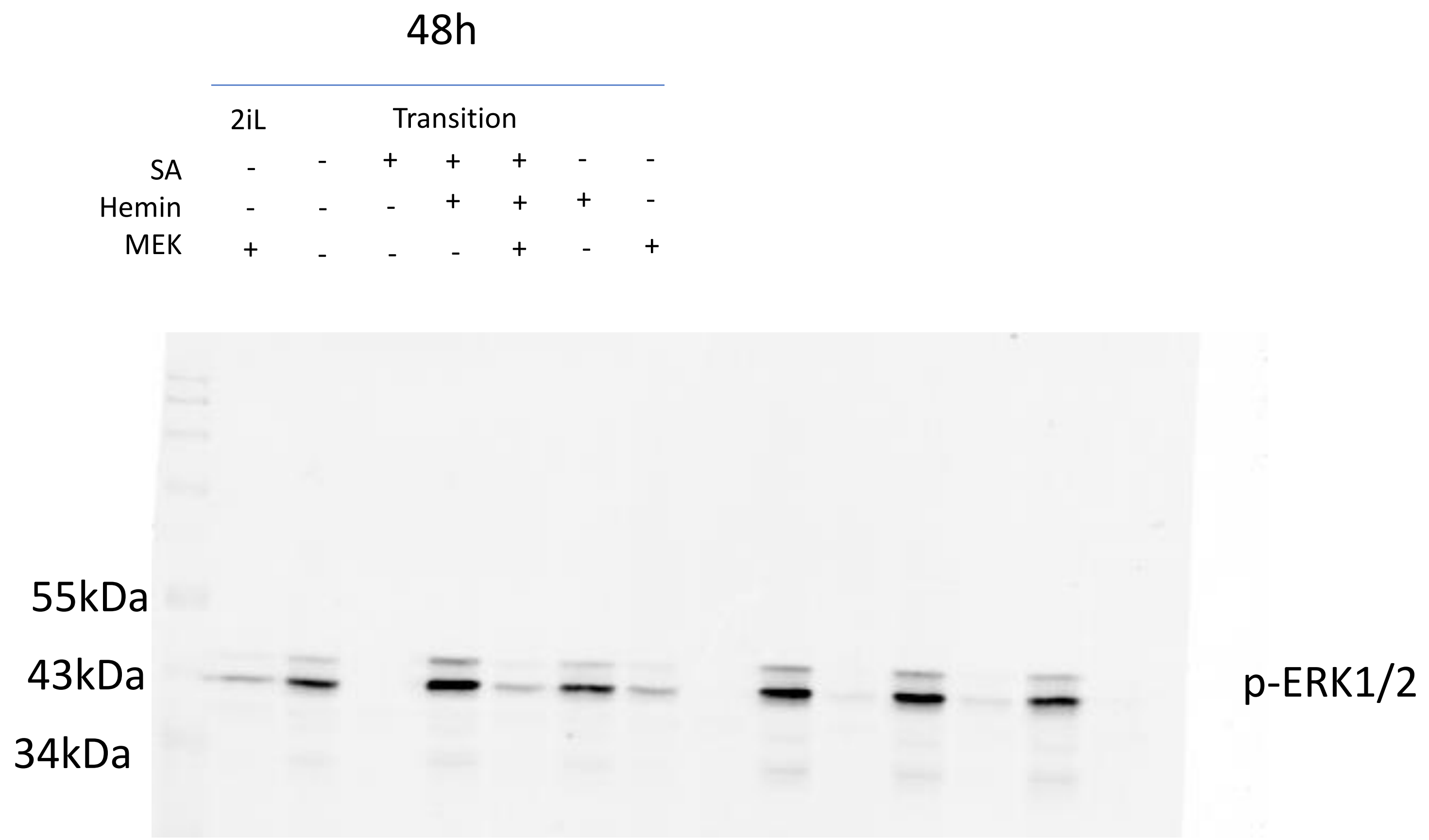
48h
2iL
Transition
SA - - + + + - -
Hemin - - - + + + -
MEK + - - - + - +
55kDa
43kDa
34kDa
p-ERK1/2

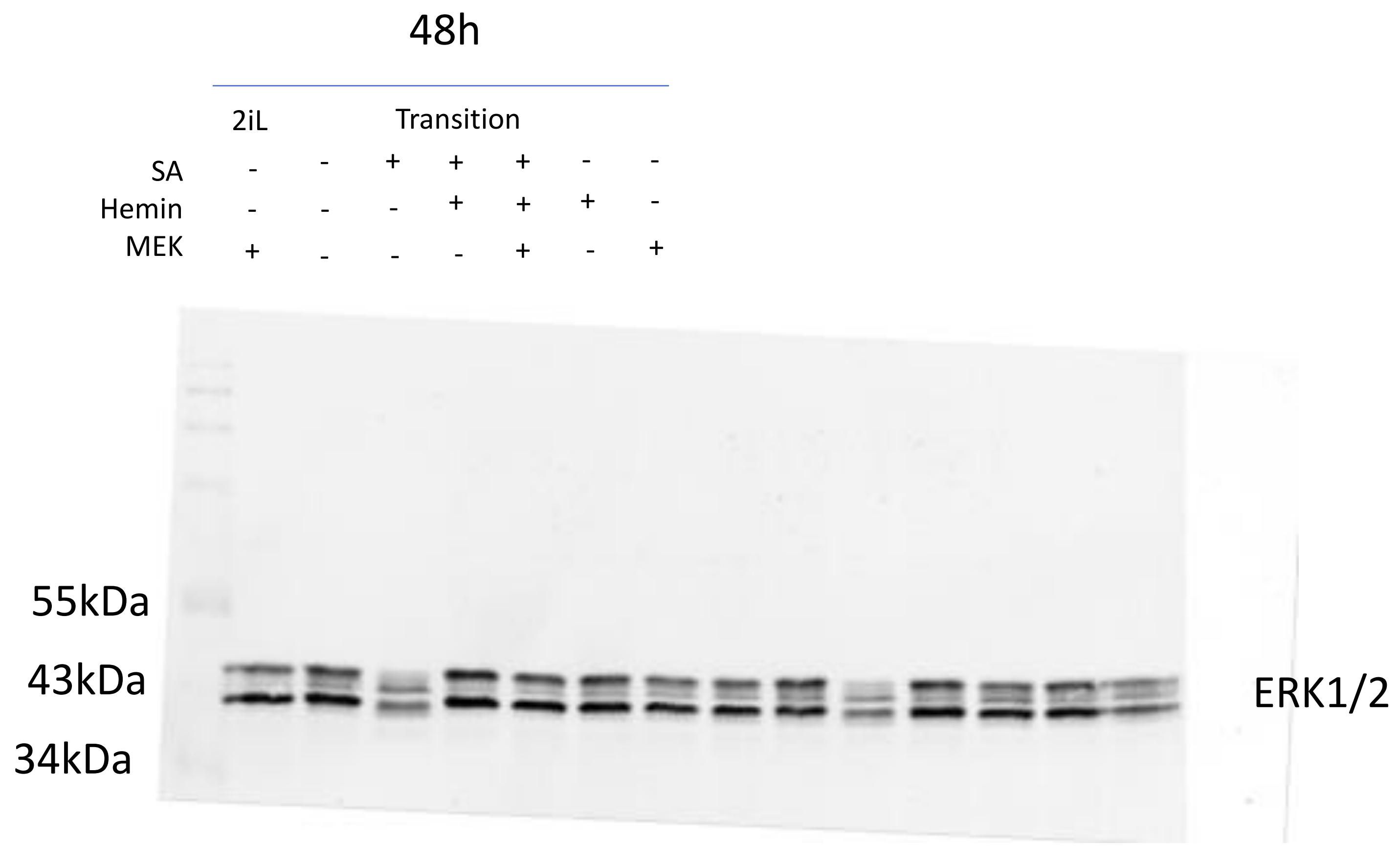
48h
2iL
Transition
SA - - + + + - -
Hemin - - - + + + -
MEK + - - - + - +
55kDa
43kDa
34kDa
ERK1/2

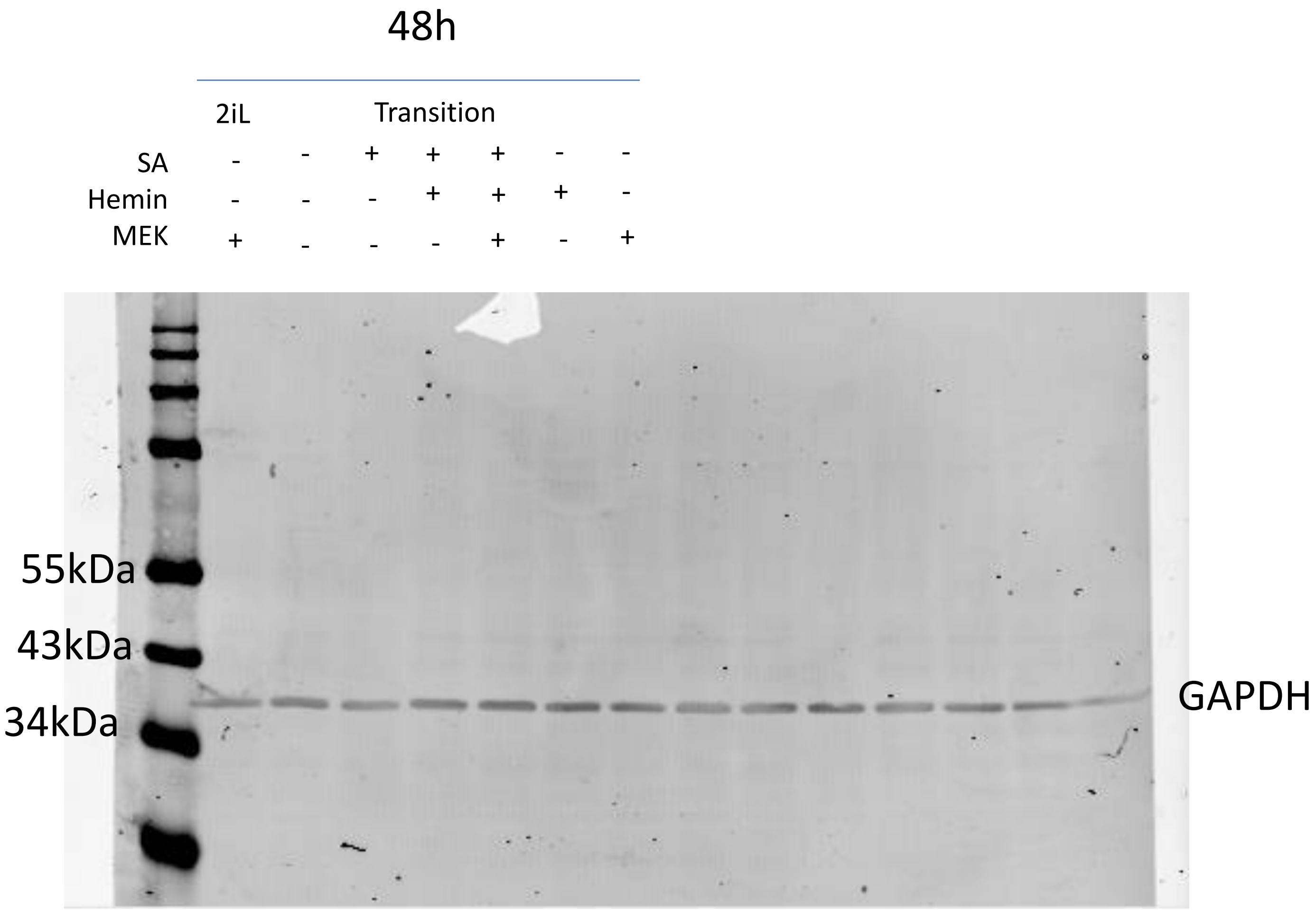
48h
2iL
Transition
SA - - + + + - -
Hemin - - - + + + -
MEK + - - - + - +
55kDa
43kDa
34kDa
GAPDH
